# Basque-Spanish Bilingual Aphasia: A Case-Study to Reveal Frequency-Based, Language-Agnostic Lexical Organization in Bilinguals

**DOI:** 10.1162/nol_a_00170

**Published:** 2025-06-23

**Authors:** Esti Blanco-Elorrieta, Miren Arantzeta

**Affiliations:** Department of Psychology, New York University, New York, NY, USA; Department of Neural Science, New York University, New York, NY, USA; Department of Linguistics and Basque Studies, University of the Basque Country (UPV-EHU), Bizkaia, Spain

**Keywords:** aphasia, bilingualism, language organization, lexical access, multilingualism

## Abstract

This study investigated whether language serves as the primary organizational axis dividing lexico-semantic representations in multilingual individuals, or whether language is a subsidiary feature to dominant organizing principles identified in monolingual individuals. To address this question, we examined the influence of two well-established principles of language organization—frequency and concreteness—on naming accuracy in a post-stroke bilingual individual with anomic aphasia (PWA). The participant, a highly proficient Basque-Spanish bilingual, underwent MRI scanning to delineate the extent and location of the lesion and completed a naming-by-definition task in both languages, along with a control group of 24 age-matched bilinguals. Stimuli were orthogonally varied by frequency (high/low) and concreteness (high/low). Generalized linear mixed models revealed main effects of both frequency and concreteness on naming accuracy. Notably, while healthy controls showed a robust concreteness effect—with concrete words yielding higher accuracy—the PWA exhibited a disproportionately larger impairment for low-frequency words. This pattern, consistent with the lesion’s location to the inferior temporal gyrus, highlights a specific vulnerability of frequency-based lexical representations following temporal lobe damage. Importantly, the bilingual PWA demonstrated strikingly similar error rates across languages, yet an item-level analysis revealed that the specific words affected differed between the two languages. These findings (i) clarify the role of the inferior temporal gyrus in lexical organization, (ii) suggest that bilinguals possess an integrated lexical system governed by general cognitive principles, and (iii) challenge the notion that language itself is the dominant axis of organization in the bilingual mind/brain.

## INTRODUCTION

Despite multilingual individuals comprising most of the world’s people, a lack of research on this population has resulted in researchers not having an answer to a fundamental question: Do bilingual individuals rely on a shared language system or is each language represented independently in the bilingual mind/brain? At its crux, answering this question requires specifying the principles of language organization.

In monolinguals, research on patients with acquired language disorders has been instrumental in shaping modern theories of the mental lexicon’s general organization, the internal structure of its components, and the arrangement of underlying semantic knowledge. This is especially evident in cases where individual linguistic units are affected, as selective impairments have been viewed as evidence of the independent organization of lexical and conceptual representations. Such impairments can arise from phonological deficits (e.g., conduction aphasia of the reproduction type), lexical deficits (e.g., anomia or transcortical sensory aphasia), or morphosyntactic disorders (e.g., agrammatism). Lexical retrieval can also be selectively impaired for specific word types. Category-specific impairments have been reported for natural categories like fruits, vegetables, and animals, as well as for artificial objects such as tools and furniture (e.g., Basso et al., 1988; Caramazza & Mahon, 2003; Hart et al., 1985; McCarthy & Warrington, 1987; Warrington & McCarthy, 1983; see Tyler et al., 2000, for an overview). Dissociations have also been noted between abstract and concrete nouns (Goldstein, 1948; Goodglass et al., 1969; Head, 1926; Mancano & Papagno, 2023), and some studies have documented selective impairments in retrieving the names of colors (Beauvois, 1982; Beauvois & Saillant, 1985) and proper names (Semenza, 2022; Semenza & Zettin, 1988, 1989). Thus, these studies have served to lay the groundwork of what features (e.g., word class, concreteness, name type) are the fundamental organizational principles underlying the human language system.

In the case of bilingual individuals, there is an outstanding question regarding the role language plays as an organizational principle. On the one hand, it could be that language is the dominant axis dividing lexico-semantic representations in the multilingual brain, such that elements belonging to Language 1 (L1) and Language 2 (L2) are separated in a substantive manner at the highest level of representation, giving rise to two parallel, independent systems (e.g., Abutalebi et al., 2001; Chee et al., 1999, 2000; Giussani et al., 2007; Green, 2003; Ou et al., 2020; Perani & Abutalebi, 2005). Models aligned with this view suggest that bilinguals can selectively inhibit one language at a time (Green & Abutalebi, 2013), an approach that presupposes that language is an axis of representation that can be selectively activated or deactivated.

Alternatively, language could be not a core organizational principle but a feature embedded within an existing organizational architecture—akin to features such as register or word class—such that (i) multilinguals would have a single language system containing elements and structures of both languages and (ii) language would be subsidiary to established, dominant organizing and selection principles (Blanco-Elorrieta & Caramazza, 2021).

These two possibilities yield vastly divergent neural predictions. The first scenario entails that each language is supported by distinct neural circuits that can be selectively deactivated, while the second suggests that both languages share common neural mechanisms. Empirical evidence from healthy individuals is mixed. Although a shared conceptual system is consistently supported (Chen et al., 2025; Van de Putte et al., 2018), the question of whether lexical and morphosyntactic processes are handled by the same mechanisms is highly contested. Extensive neuroimaging research (Hasegawa et al., 2002; Meykadeh et al., 2021; Ou et al., 2020; Rüschemeyer et al., 2006) shows varying results: Some studies report overlapping activation for L1 and L2 (Perani & Abutalebi, 2005; Sakai et al., 2004; Tettamanti et al., 2002; Yang et al., 2017), while others find distinct patterns—with increased activation for either L1 (Lehtonen et al., 2009) or L2 (Golestani et al., 2006; Hasegawa et al., 2002; Ip et al., 2017)—and some report overlapping yet language-specific activations across regions of interest (ROIs; Gao et al., 2023; Meykadeh et al., 2021; see meta-analysis in Cargnelutti et al., 2019 and Biondo et al., 2023, for review).

Importantly, neuropsychological evidence can more conclusively test these models. If language is indeed the feature that dominates the organizational space, one would expect language-selective deficits at different levels of representation. For instance, it should be possible to observe deficits in one language but not the other, or in a specific feature (e.g., word class) within one language that does not generalize to the other. Under this account, it would be statistically unlikely to observe qualitatively equal deficits across both languages of a multilingual individual. If language is instead a subsidiary feature to the organizational principles in place for monolingual individuals, then one would expect that impairments in any one component of this lexico-semantic organization (e.g., frequency, animate objects, tools) should be equally distributed across a balanced multilingual person’s languages. It is important to note here that the predictions would diverge for balanced and unbalanced multilinguals. To the extent that stronger representations are more resistant to damage, in the case of an unbalanced bilingual, we should expect more of the weaker representations (L2) to be affected than L1. However, the critical point here is that this would be a *quantitative*, and not a *qualitative* difference (i.e., we should expect the same kind of deficits across languages, even if they manifested to different degrees).

Here, we turned to a 24-year-old Basque-Spanish early balanced bilingual with post-stroke anomic aphasia to tease apart these two possibilities by independently evaluating the cross-linguistic influence of two well-established organizational principles: frequency and concreteness.

In psycholinguistic studies, *concreteness* is often used to describe a word or sentence’s ability to evoke visual imagery (e.g., Jonides et al., 1975). Generally, words like “dog” have high visual concreteness because they refer to tangible objects. In contrast, words such as “loyalty” or “justice” do not strongly prompt the creation of visual mental images. Concreteness is an ideal variable to test our hypotheses because a convergent body of evidence from neuroimaging and neuropsychology has revealed it as a key organizational principle of the language system. For once, the influence of concreteness on word processing is well established in the literature, with concrete words being processed more accurately and efficiently than abstract words in healthy adults (e.g., Bottini et al., 2022; Khanna & Cortese, 2021; Wiemer-Hastings & Xu, 2005) and people with aphasia (e.g., Hoffman et al., 2011; Papagno, 2022; Sandberg & Kiran, 2014). For another, numerous neuroimaging studies have compared concrete, highly imageable words with abstract, less imageable words (e.g., Bedny & Thompson-Schill, 2006; Hauptman et al., 2022; Hoffman et al., 2015; Mestres-Missé et al., 2009; Montefinese et al., 2021; Pexman et al., 2007; Sabsevitz et al., 2005) and have found that concrete words selectively recruit visual-related brain regions, suggesting separate cortical representations for concrete and abstract entities (see Miceli et al., 2022; see Binder et al., 2016, for a review). In terms of neuropsychology, there is a long tradition of documenting dissociations between abstract and concrete nouns, and in fact, these dissociations were among the first to ever be documented (Goldstein, 1948; Goodglass et al., 1969; Head, 1926). These category specific naming impairments have been explained as the result of a different form of organization of the underlying conceptual knowledge.

According to Paivio’s (1990) dual coding theory concrete words are stored and accessed through both verbal and visual associations, while abstract words rely solely on verbal associations. In the case of bilinguals, Paivio and Desrochers (1980) argued that both concrete and abstract words have verbal representations in L1 and L2, but only concrete words have an additional imagery reference, shared by translation pairs in both languages. Consequently, concrete words can be translated directly between the two verbal systems or indirectly through the shared image system, whereas abstract words can only be translated between the verbal systems. If language is a high-order organizational principle of the lexical system, one would predict an interaction whereby only concrete words could show equal loss across languages (presumably due to damage to the unified imagery system). Abstract words, which are purely dependent on verbal systems, could be (i) spared, or (ii) if they were to be impaired, there would be no grounds to expect this impairment to be equal across languages. If bilinguals have a unified language system on the other hand, one expects the effect of concreteness to be qualitatively equal across languages.

The second property that this study will evaluate is Frequency. Frequency refers to how often a word appears in a language, typically measured in written or spoken texts. Words that are encountered frequently, like “the” or “and,” are considered high-frequency words, while words that appear less often, such as “serendipity” or “quintessential,” are low-frequency words. Word frequency effects have been taken to be a marker of lexical access in healthy individuals (Balota & Chumbley, 1984; Jescheniak & Levelt, 1994; Oldfield & Wingfield, 1965; Schmidtke et al., 2025), and monolingual aphasic patients have been shown to hesitate more and make more errors on low-frequency as compared to high-frequency words (Gagnon et al., 1997; Gordon, 2002; Mack et al., 2015; Schwartz et al., 2004). Importantly, unlike concreteness, which taps onto the semantic representations of words, word frequency effects are tightly related to the retrieval of the phonological form (Jescheniak & Levelt, 1994). Given the purely lexical nature of this feature, it is not obvious the extent to which it should generalize across languages. Under the hypothesis that lexical items are organized at the highest order by language, and provided that the possibility of a deficit in shared semantic representations is ruled out, two separate frequency distributions would have to be individually lesioned on the same end to show equal impairment across languages. Thus, only under the hypothesis that bilinguals have a single lexical system should one expect potential frequency-related deficits to be qualitatively equal across languages.

The frequency predictions relate tightly to expected cognate/cross-language homophone effects. If homophones share a common word-form representation (Barr et al., 2022; Jescheniak & Levelt, 1994), we expect cognates to be less impaired than non-cognates, as their cumulative frequency across languages should result in higher overall frequency than non-homophones. Instead, a lack of cognate effect would support a model where cross-language homophones have fully independent representations (e.g., Caramazza et al., 2001).

Last, this study also aims to establish a causal relationship between the anatomical location of the lesion and potentially observed deficits. Numerous neuroimaging studies using functional magnetic resonance imaging (fMRI), positron emission tomography, and transcranial magnetic stimulation have explored the role of concreteness and frequency on the neural underpinnings of lexical representations. Regarding the effect of concreteness, studies consistently highlight the involvement of the left inferior frontal gyrus and other language-related regions, such as the left superior temporal gyrus and the temporal pole, in processing abstract words compared to concrete words in healthy adults (e.g., Bucur & Papagno, 2021; Goldberg et al., 2007; Hoffman et al., 2010, 2015; Perani et al., 1999; Sabsevitz et al., 2005; Sánchez et al., 2023). In contrast, processing concrete words has been found to engage temporal regions, including the posterior inferior temporal gyrus (ITG), medial anterior temporal lobe, and left inferior temporal pole (Binder et al., 2005; Noppeney & Price, 2002; Sabsevitz et al., 2005). These findings align with the view that concrete concepts are supported by temporal and occipital regions involved in sensory processing and visual object recognition, while abstract concepts depend more heavily on frontal areas associated with semantic-executive control (Breedin et al., 1998; Hoffman et al., 2015; Noppeney & Price, 2002).

Regarding the neural correlates of frequency, previous research has found that low-frequency words evoke greater activation compared to high-frequency words in left prefrontal regions (e.g., Carreiras et al., 2006, 2009; Chee et al., 2003; Fiebach et al., 2002, 2003; Nakic et al., 2006; Protopapas et al., 2016; Sánchez et al., 2023) and in the inferior temporal cortex, particularly the left fusiform gyrus (e.g., Bruno et al., 2008; Chee et al., 2003; Hauk et al., 2008; Joubert et al., 2004; Keller et al., 2001; Kronbichler et al., 2004). However, most of these studies have varied either frequency or concreteness without controlling for the other variable (e.g., Carreiras et al., 2006; Liu et al., 2008; Weissbart & Martin, 2024). Consequently, given the high correlation between both variables (i.e., concrete words have typically higher frequencies) these studies have not always been able to isolate the neural correlates of each of these variables independently.

In all, this study targets three empirical questions with deep theoretical implications. First, it aims to establish whether frequency and concreteness independently affect lexical production in aphasia. Second, it aims to establish a relation between the location of the lesion and the observed deficits to provide a causal link between brain anatomy and function. In other words, we aim to establish a one-to-one mapping to assess what lexical functions this area is *necessary* for. Third, it aims to evaluate whether potential deficits in either of these dimensions evenly affect a bilingual’s languages to further our understanding of language organization in the multilingual brain.

## MATERIALS AND METHODS

### Participants

#### Person with aphasia

The study focused on a single person with aphasia (PWA), a 24-year-old right-handed woman with a university education. She had a hemorrhagic stroke due to a cerebral arteriovenous malformation rupture, which resulted in a left temporal intraparenchymal hematoma. The individual is a highly proficient early balanced L1-Basque and L2-Spanish bilingual. On the Language Experience and Proficiency Questionnaire (LEAP-Q; Marian, Blumenfeld, & Kaushanskaya, 2007, adapted to Basque-Spanish), the patient scored as follows: Composite Factor Score: 13.91 (range −6 to 32), Basque Home Use and Proficiency: 12.99 (range −13 to 24), and Basque Social Use: 31.75 (range −7 to 80). Scores closer to the midpoint of each range represent a more balanced bilingual profile, with higher values indicating greater Basque dominance and lower values reflecting greater Spanish dominance. The patient’s Composite Factor Score of 13.91 reflects high proficiency and balanced use of both Basque and Spanish. She received formal education in Basque until university, where she pursued a university degree in Spanish.

An evaluation of language, executive functions (including inhibitory control, cognitive flexibility, and processing speed), and memory was conducted 2 months post-stroke. Language assessment was conducted in both Basque and Spanish in separate sessions using the Bilingual Aphasia Test (Paradis & Libben, 1987; adapted to Basque-Spanish bilinguals by Erriondo et al., 1989), Boston Naming Test (Kaplan et al., 2005), Action Naming Battery (Druks & Masterson, 2000), and Pyramids and Palm Trees Test (Howard & Patterson, 1992), as well as semantic and phonological verbal fluency tasks. Executive functions and memory abilities were assessed using the Color-Word Stroop Test (Golden, 2010), the Modified Wisconsin Card Sorting Test (M-WCS; Nelson, 1976; Schretlen, 2010), the TESEN (Trail-Making Test; Gil & del Ser, 2011), and the Wechsler Memory Scale-IV (WMS-IV; Wechsler, 2012). Since there are no validated Basque versions of these tests, these assessments were conducted in Spanish. The raw scores of the participant were corrected using European Spanish norms based on large population samples, accounting for the individual’s age and education level. This process allowed us to derive standardized scores, such as T scores, percentiles, and decatypes, depending on the specific test used. She was diagnosed with anomic aphasia affecting both L1 and L2 equally, as shown in Table 1. She presented preserved executive function abilities and inhibitory control but significant memory impairment across all domains (see Table 2). Detailed data from the complete assessment are provided in Supplementary Material 3, available at https://doi.org/10.1162/nol_a_00170.

**Table 1.** Word retrieval and verbal fluency abilities 2 months post-onset

Word retrieval and fluency abilities	Basque raw score (% correct)	Spanish raw score (% correct)
Boston Naming Test	20 (33%)	28 (47%)
Action Naming Test	50 (50%)	57 (57%)
Phonological Fluency*	9	8.33
Semantic Fluency**	3	3.5

* Phonological Fluency (1 min): Basque phonemes b/k/l, Spanish phonemes p/f/k.

** Semantic Fluency (1 min): Categories “animals” and “fruits.”

**Table 2.** Cognitive test performance 2 months post-stroke

Executive functions and memory	T score
Word-Color STROOP	
Word	48
Color	41
Word-Color	43
Interference control	46
TESEN (Trail-Making)***	
Execution	45
Speed	45
Precision	45
M-WCS	
Complete categories	51
Perseverative errors	52
Total errors	51
% perseverative	47
Exec. Index	51
WMS-IV***	
Auditory memory	19
Visual memory	31
Visual working memory	36
Immediate memory	23
Delayed memory	19

*Note*. In the assessment of executive functions and memory abilities, T scores represent standardized scores adjusted for age, education, and/or sex.

*** For the TESEN (Trail-Making) and WMS-IV tests, T scores were derived from provided percentiles using a standard conversion formula (T = 50 + 10 × *z*), where *z* scores were calculated based on the normal distribution.

#### Control group

The non–brain-damaged (NBD) group comprised 24 university students (*M* = 21.8 years; *SD*: 1.95). All participants were early Basque L1 and Spanish L2 bilinguals. They were highly proficient in both languages, with self-reported Basque proficiency of 9.72/10 (*SD* 0.39) and Spanish proficiency of 9.35/10 (*SD* 0.51). Their daily language usage was 52.39% Basque (*SD* 12.14) and 35.4% Spanish (*SD* 13.83). None of the control participants had a history of neurological or psychiatric disorders, and all reported normal or corrected-to-normal vision and hearing.

The study was approved by the Ethics Committee for Research Involving Human Beings, Their Samples, and Their Data (CEISH) of the University of the Basque Country under the code: M10_2023_395. All participants were fully informed about the study, provided written consent, and the research was conducted in accordance with the principles of the Declaration of Helsinki.

### Materials

A total of 180 nouns were selected for the experimental task and used in both Basque and Spanish, resulting in 360 stimuli. The stimuli were controlled by two key variables: frequency and concreteness. These variables were orthogonally crossed, resulting in four distinct experimental conditions: 45 high-frequency concrete nouns, 45 low-frequency concrete nouns, 45 high-frequency abstract nouns, and 45 low-frequency abstract nouns in each language. Fifteen nouns in each of the four categories were cognates between Basque and Spanish.

Concreteness norms were taken from the database by Brysbaert et al. (2014), which provides concreteness ratings for 40,000 English word lemmas. Following their criteria, concrete nouns are those with values above 4 on a 1–5 scale, and abstract nouns have values below 2.3. Lemma frequency in Spanish was obtained from the EsPal database (Duchon et al., 2013), while frequency values in Basque were sourced from the EHME database (Acha et al., 2014). Nouns with a log lemma frequency below 1.5 were classified as low frequency, whereas frequent nouns had a log lemma frequency above 1.5. There were no significant differences in word length between the two languages (*M*_(Sp)_ = 7.01 phonemes, *M*_(Bsq)_ = 7.13; *t*_(356)_ = 0.42, *p* = 0.66).

We created definitions for each noun in Basque and Spanish. Definitions were longer in Spanish than in Basque, primarily because Spanish has free-standing function words, whereas Basque is an agglutinative language. This difference was statistically significant (*t*_(345)_ = −10.491, *p* < 0.001), with a mean definition length of 12.50 words in Basque and 16.95 words in Spanish. However, the auditory duration of the definitions did not vary significantly across the four experimental conditions (*F*_(3, 355)_ = 0.41, *p* = 0.74). All the materials used in the study are provided in the Supplementary Materials 1 and 2.

### Design and Procedure

The experiment consisted of a naming-by-definition task (e.g., Kiran & Tuchtenhagen, 2005) in which participants were provided with the definition of an item and required to generate the corresponding lexical term. The stimuli were presented randomly across the four experimental conditions: high-frequency concrete nouns, low-frequency concrete nouns, high-frequency abstract nouns, and low-frequency abstract nouns.

Participants were presented with the stimuli auditorily and were allowed to listen to them as many times as needed. No time limit was set to provide an answer. The individual with aphasia completed the experiment in a face-to-face setting with two independent experimenters, both native Basque-Spanish speakers, while control participants completed the task using the online platform Qualtrics. The auditory stimuli for the Qualtrics platform were recorded at a constant pace by one of the experimenters who conducted the face-to-face sessions with the PWA. Recent studies show that people obtain equivalent scores online versus in-person (e.g., Chan & Ahn, 2023; Uittenhove et al., 2023), and we are thus confident that the administration mode did not influence the results.

The experiment was conducted over two independent sessions for control participants and four independent sessions for the individual with aphasia. Each language (Basque and Spanish) was tested in separate sessions, and to address any potential sequence effect the language order was counterbalanced across sessions and participants. There was a minimum interval of 7 days between sessions.

### Data Analysis

#### Neuroimaging data

**Figure 1.**
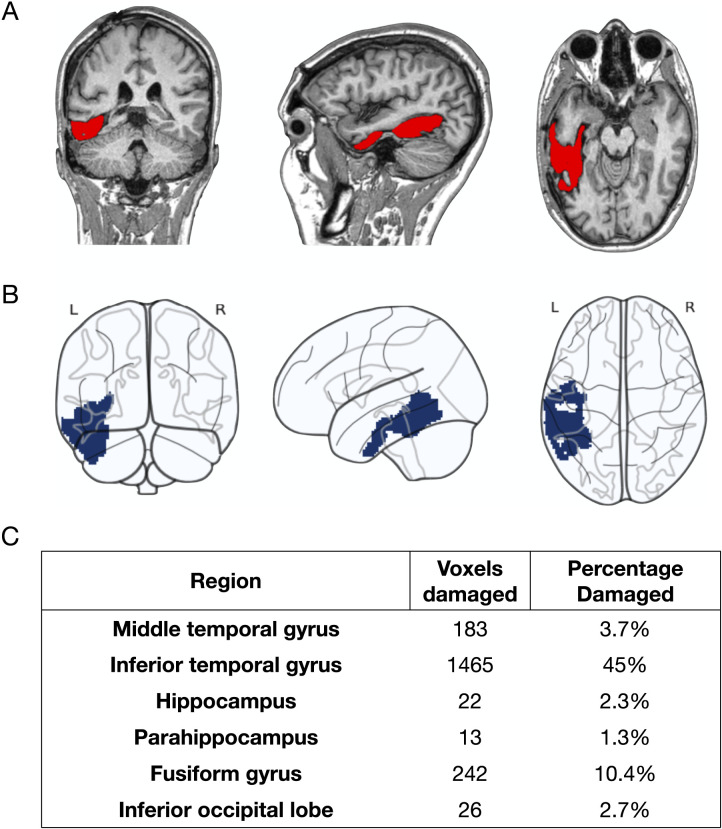
Extent and location of PWA’s lesion. (A) Shows lesion traced in native T1 contrast. (B) Shows lesion projection onto MNI space. (C) Shows quantification of the lesion. PWA = person with aphasia, MNI = Montreal Neurological Institute.

The patient’s MRI scan was performed on a clinical 1.5T MRI scanner (Ingenia; Philips Healthcare, the Netherlands) at the Osatek Center in Donostia—San Sebastián. The T1-weighted structural scan was acquired in a 3D TFE (Turbo Field Echo) sequence in 231 sagittal slices with 1 mm isotropic voxels (repetition time [TR]: 7.7 ms; echo time [TE]: 3.6 ms; field of view [FoV]: 320 × 320). To create a lesion mask in the native space, we identified the lesion site and drew the lesion mask on the T1w image using the MRIcron software. The lesion mask was first drawn with the 3D tool and then manually corrected slice by slice. The resulting lesion mask was smoothed with a 4 mm full width at half maximun (FWHM) factor. To standardize the images for further analysis, we registered both the T1w image and the lesion mask to the MNI space, a widely used standardized brain template developed by the Montreal Neurological Institute. The MNI space allows for spatial normalization, meaning that individual brain scans can be aligned with a common reference brain to facilitate comparisons across subjects or studies. To register the T1w image and the lesion mask to the MNI space, we used the SPM12 software (Ashburner et al., 2014), which applies tissue probability maps (TPM) to improve alignment and incorporates the lesion mask as an additional TPM. The calculated transformation was applied to both the T1w image and the lesion mask to transform them to the MNI space. Once in MNI space, we calculated the number and the percentage of voxels damaged in each ROI in the AAL atlas (Rolls et al., 2020) using the Nilearn package (Abraham et al., 2014) in Python. The lesion mostly affected the inferior temporal and fusiform gyri (Figure 1).

#### Behavioral data

To ensure the reliability and accuracy of the results, a meticulous review process was undertaken for the participants’ responses, which were manually evaluated by a native speaker of Basque and Spanish. Only the target answers or acceptable dialectal variations were marked as correct. Responses that were semantically related but not exact matches (e.g., hypernyms, hyponyms) were not considered accurate, along with any other responses that did not meet the precise criteria.

An overview of performance trends across groups and conditions is presented in Table 3. These descriptive statistics summarize correct response rates across frequency (high/low) and concreteness (high/low) conditions in both Basque and Spanish, separately for the PWA and the NBD group. This preliminary overview highlights performance differences between groups, with notable variations across language, frequency, and concreteness conditions.

**Table 3.** Accuracy percentages by frequency and concreteness conditions in Basque and Spanish for PWA and NBD groups

Frequency	Concreteness	Language	PWA (%)	NBD (%; *SD*)
High	High	Basque	84.4	94.6 (3.8)
High	High	Spanish	86.7	95.3 (3.7)
High	Low	Basque	46.7	81.2 (9.0)
High	Low	Spanish	48.9	81.8 (8.0)
Low	High	Basque	40.0	92.4 (5.4)
Low	High	Spanish	37.8	92.8 (4.6)
Low	Low	Basque	17.8	73.0 (10.0)
Low	Low	Spanish	17.8	73.6 (7.9)

*Note*. Percentages indicate correct responses for PWA and average accuracy for non–brain-damaged (NBD), accompanied by standard deviation (*SD*).

To evaluate the effects of frequency and concreteness on participants’ lexical retrieval, as well as the extent to which language deficits were equal or unequal across languages, data were analyzed using generalized linear mixed models (GLMMs) with a binomial distribution, appropriate for modeling accuracy data (correct/incorrect). Random effects, fixed effects, and their interactions were introduced progressively into the models, with their goodness of fit assessed using the Akaike information criterion (AIC; Akaike, 1974). A reduction of 2 points in the AIC was considered evidence of improved model fit, and variables that contributed to this improvement were retained in the final model. The fixed effects included frequency (high/low), concreteness (high/low), group (NBD/PWA), language (Basque/Spanish), word length (in phonemes), and cognate status (cognate/non-cognate). However, due to convergence issues, cognate status could not be included in the final model.

The final model included the main effects of frequency, concreteness, and group, as well as the interaction between frequency and group. The random effect of target accounted for the variability in accuracy across different items. Estimated marginal means (EMMs) for the main effects and interactions were calculated using the emmeans package (Lenth, 2025), which provides adjusted predicted values by accounting for other variables in the model, thereby enabling clearer comparisons across conditions. Pairwise comparisons, adjusted for multiple testing using Tukey’s HSD (honestly significant difference), were conducted to examine differences between specific factor levels. Additionally, we conducted a contrast analysis to further explore how the effect of frequency varied between the NBD and PWA groups. The analyses were conducted using R (Version 4.3.0) and the lme4 package (Bates et al., 2015) for model fitting, with additional analyses performed using the emmeans package.

Last, we performed a Cohen’s kappa analysis on the PWA’s error distributions in Basque and Spanish. Cohen’s kappa is a statistical measure that evaluates the agreement between two distributions beyond what would be expected by random chance. This comparison was crucial because while an equal number of errors across languages would align with the theoretical perspective that language is secondary to broader organizational principles, it could stem from two distinct sources: semantic or lexical.

On the one hand, if semantic representations are not divided by language and are instead shared across languages (see also Goldrick et al., 2016; Pyers & Emmorey, 2008; Spalek et al., 2014; Starreveld et al., 2014), then equal errors across both languages could be the consequence of damage to these shared representations for a given concept. This possibility would reflect a semantic, not lexical, deficit. On the other hand, equal number of errors could stem from damage to language agnostic lexical distributions in the bilingual mind/brain. In other words, under the view that language is not the dominant axis dividing the lexical system, elements of both languages would be intermixed in every lexical category (high/low frequency, animate/inanimate, etc.). Thus, any deficit affecting any one lexical category (e.g., high/low frequency) should manifest similarly across both languages of a highly balanced bilingual PWA.

Here, a high kappa score would mean that the exact same items were impaired in Spanish and Basque, pointing to the underlying semantic representations shared by both languages being impaired. Conversely, a low or negative overlap would indicate that the errors in low-frequency items stemmed from deficits in processing the lower frequency ends of the distribution, rather than damage to shared conceptual representations.

## RESULTS

**Figure 2.**
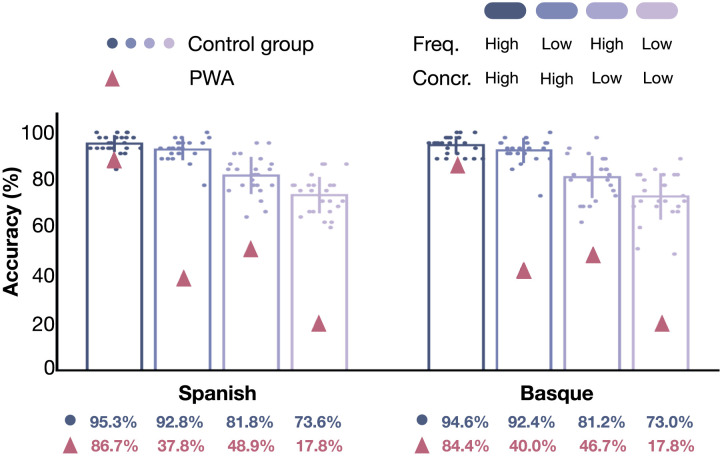
Accuracy (%) of naming-from-definition tasks, categorized by frequency (high/low) and concreteness (high/low) in Basque and Spanish languages. Bars represent group mean accuracy for each category, with vertical lines at the top of each bar indicating the standard error, while the overlaid dots indicate individual participant means.

The regression analyses revealed significant main effects of Frequency, Concreteness, and Group on naming accuracy, as well as a notable interaction between Frequency and Group. These results are illustrated in Figure 2, which shows the percentage accuracy by frequency, concreteness, and group in each language.

Estimated marginal mean accuracy was higher for high-frequency words (*M* = 1.85, *SE* = 0.14, 95% CI [1.58, 2.11]) compared to low-frequency words (*M* = 0.42, *SE* = 0.13, 95% CI [0.16, 0.67]), with a pairwise comparison indicating a significant difference of 1.43 (*SE* = 0.19, *z* = 7.63, *p* < 0.001). Similarly, accuracy was greater for high-concreteness words (*M* = 1.98, *SE* = 0.13, 95% CI [1.73, 2.22]) than for low-concreteness words (*M* = 0.29, *SE* = 0.11, 95% CI [0.06, 0.51]), with a significant difference of 1.69 (*SE* = 0.15, *z* = 11.35, *p* < 0.001). Group differences were also evident, with the NBD group demonstrating higher accuracy (*M* = 2.41, *SE* = 0.08, 95% CI [2.26, 2.57]) than the PWA (*M* = −0.15, *SE* = 0.15, 95% CI [−0.44, 0.14]), and a significant contrast of 2.57 (*SE* = 0.14, *z* = 17.84, *p* < 0.001).

**Figure 3.**
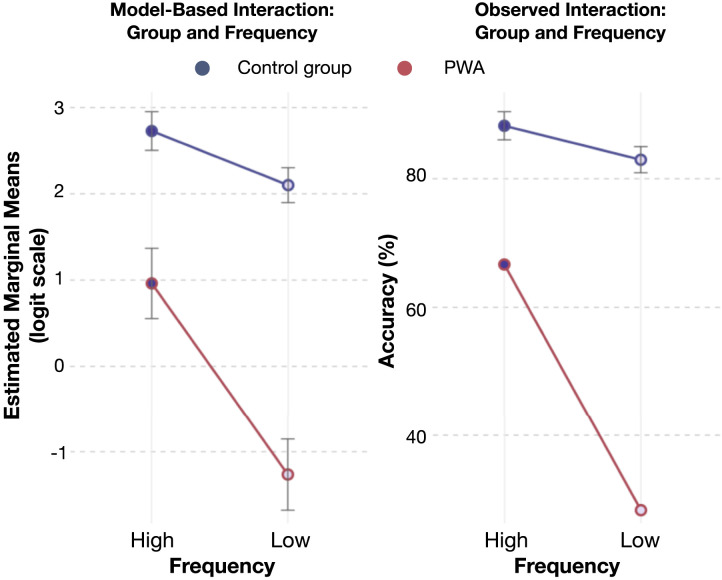
Interaction between frequency and group on naming-by-definition accuracy. The left plot shows estimated marginal means on the logit scale with 95% confidence intervals. The right plot shows observed accuracy percentages with 95% confidence intervals for the NBD group (averaged) and single data points for the PWA group. The PWA group shows a larger drop in accuracy for low-frequency words.

The interaction between frequency and group indicated that the effect of frequency varied across groups. In the NBD group, the estimated marginal means were 2.73 (*SE* = 0.11, 95% CI [2.50, 2.95]) for high frequency and 2.10 (*SE* = 0.10, 95% CI [1.90, 2.30]) for low frequency, with a difference of 0.63 (*SE* = 0.15, *z* = 4.23, *p* < 0.001). In contrast, in the PWA, the means were 0.96 (*SE* = 0.21, 95% CI [0.56, 1.37]) for high frequency and −1.26 (*SE* = 0.21, 95% CI [−1.68, −0.85]) for low frequency, with a larger difference of 2.23 (*SE* = 0.30, *z* = 7.50, *p* < 0.001). A contrast analysis was performed to further examine this interaction, confirming that the effect of frequency was significantly more pronounced in the PWA than in the NBD group, with an estimated contrast of −1.60 (*SE* = 0.28, *z* = −5.64, *p* < 0.001; see Figure 3).

**Figure 4.**
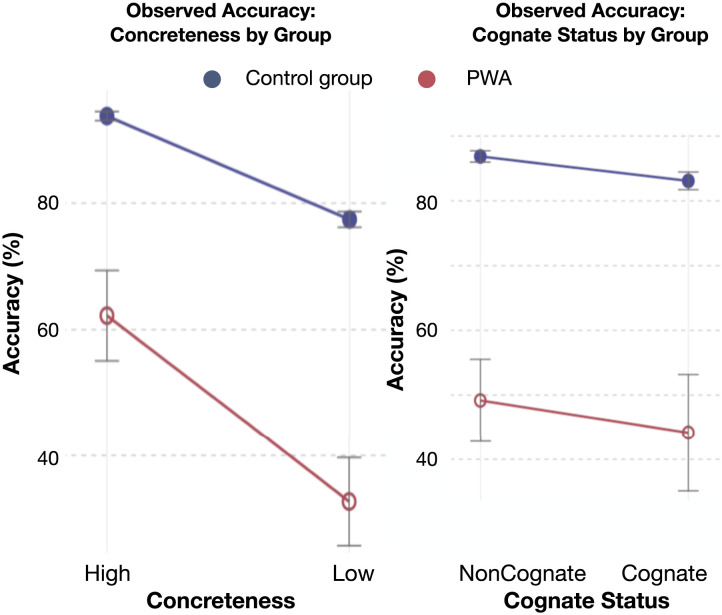
Effects of concreteness (left) and cognate status (right) on accuracy averages on a naming-by-definition task. Accuracy is expressed as the proportion of correct responses out of total trials per condition. The plots show observed accuracy percentages with 95% confidence intervals, with group means for the NBD group and individual data points for the PWA group.

Additionally, we provide visualizations for the effects of Concreteness and Cognate Status on Figure 4. For the reasons stated above, neither of these effects could be included in the final statistical model, but they are included here to offer a broader descriptive perspective on how both factors relate to naming accuracy in PWA and NBD participants.

Cohen’s kappa analysis on the error distribution was −0.206 (*p* = 0.027), indicating that the observed agreement was lower than would be expected by random chance. In other words, even though the PWA made significantly more errors in low-frequency items in both languages, it was different items that the PWA failed to name in each language. This suggests that the source of the PWA’s deficit is unlikely to be due to damage to shared semantic representations and it is rather a lexical deficit on the lower end of a shared lexical distribution.

## DISCUSSION

This study set out to address three independent questions. First, it aimed to establish whether frequency and concreteness independently affect lexical production in aphasia. Second, it sought to establish a causal relationship between the anatomical location of the lesion and the observed deficits. Third, it aimed to establish whether selective deficits in either frequency or concreteness affect a bilingual’s languages evenly.

The results showed that, in line with previous literature, both frequency and concreteness affect lexical retrieval accuracy, even in healthy individuals. Specifically, in healthy controls, lower frequency and concreteness led to more errors. The lesion in the PWA was located primarily in the ITG and the fusiform gyrus, and it resulted in significantly lower naming scores across the board, but it was the effect of frequency that was significantly more pronounced than in controls. Importantly, this greater deficit in naming low-frequency words was perfectly equal across languages. However, the specific words impaired were different in each language. In other words, while the number of low-frequency words affected was equal across languages, the exact items differed. This suggests that the observed deficit did not emerge at the semantic level from damage to shared conceptual representations, but rather it was a deficit that affected lexical structures organized independently of language membership. In what follows we place these results within the broader literature.

The differential impact of frequency and concreteness on naming accuracy observed in this study provides critical insights into the neurocognitive mechanisms underlying word retrieval, particularly in cases of aphasia resulting from temporal lobe damage. While healthy bilingual controls exhibited a strong concreteness effect, consistent with previous studies showing that concrete words are processed more efficiently due to their richer semantic and sensorimotor representations (Binder et al., 2005; Hoffman et al., 2015; Noppeney & Price, 2004), the post-stroke bilingual individual with anomic aphasia showed a disproportionately greater impairment for low-frequency words. This finding aligns with prior research implicating the ITG in lexical frequency effects (Chee et al., 2003; Kronbichler et al., 2004; Rundle et al., 2018) and suggests that damage to this region selectively disrupts frequency-based lexical access.

Importantly, the greater vulnerability of low-frequency words compared to abstract words in the PWA challenges the assumption that concreteness effects dominate lexical processing in all cases. Instead, this pattern suggests that frequency effects may be more dependent on the integrity of phonological access mechanisms, while concreteness effects primarily emerge from differences in semantic representation (Gagnon et al., 1997; Jescheniak & Levelt, 1994). Since low-frequency words require greater phonological activation for successful retrieval and are more vulnerable to disruption (Balota & Chumbley, 1984; Fiebach et al., 2002), the disproportionate frequency-based impairment in the PWA is consistent with models positing that the ITG plays a central role in supporting access to less frequently activated lexical items. This supports prior research showing that frequency effects are tightly linked to phonological retrieval processes (Caramazza et al., 2001; Jescheniak & Levelt, 1994), whereas concreteness effects are more reliant on distributed semantic networks that include visual and motor-related regions (Binder et al., 2005; Sabsevitz et al., 2005).

Notably, much of the previous literature has not clearly separated the effects of frequency and concreteness, as these two factors tend to be correlated—concrete words are typically more frequent than abstract words (Brysbaert et al., 2014; Carreiras et al., 2006). Consequently, studies that have reported a general advantage for concrete words may have, in part, been capturing frequency effects rather than purely semantic differences. By orthogonally manipulating these two factors, our study provides evidence that frequency and concreteness exert distinct influences on lexical retrieval and that temporal lobe lesions may primarily affect frequency-dependent mechanisms rather than conceptual-semantic organization. This observation also has implications for clinical interventions: Rehabilitation strategies for aphasia may need to prioritize lexical frequency as a key factor influencing word retrieval success, particularly in individuals with ITG damage.

Since our PWA participant demonstrated similar effects across both languages—effects not attributable to damage in shared conceptual representations—this implies that language itself is not a primary organizing feature of the mental lexicon. Instead, it suggests that the cortical organization of the lexicon may be governed by resting activation levels (McClelland & Elman, 1986) that are agnostic to language membership. For balanced bilinguals, whose languages are likely to have comparable distributions of high- and low-frequency words, this would result in an equal number of impaired items across languages when low-frequency lexical items are affected. In unbalanced bilinguals, however, where the less-dominant language is often less frequently used (Nadeau, 2019), deficits that may seem selective to the non-dominant language (for an in-depth review see Kuzmina et al., 2019) may in fact reflect a frequency-based impairment.

This possibility raises an intriguing question: If cortical organization in the ITG is indeed based on frequency-related resting activation levels, could experience-driven changes in word frequency lead to cortical reorganization? For instance, if an individual transitions from a profession as a neuroscientist to a forest ranger, previously high-frequency terms like “neuron” and “inferior temporal cortex” might shift to low-frequency status, while words like “robin,” “sparrow,” and “beech tree” become more frequent. Should one expect that this change in resting activation levels would alter how and where words are processed in the inferior temporal cortex (i.e., the ITG will at some point start processing the now low-frequency “neuron,” or “inferior temporal cortex” where it didn’t before)?

This is a particularly interesting question in the case of bilingual individuals, as it is perhaps easier to illustrate the point. To the extent that words in one’s dominant language are higher in frequency, one would expect the left inferior temporal cortex to be more involved in the retrieval of L2 words. However, if one were to change their linguistic reality (i.e., by moving to a different country and stopping the use of the language of birth), under the hypothesis of activation-based cortical organization, this should shift *where* in the inferior frontal gyrus words of each language are processed.

This hypothesis is particularly compelling because it could account for differential patterns of activation found in unbalanced L1 and L2 bilinguals. For instance, Marian, Shildkrot, et al. (2007) found that despite large overlap in cortical networks activated during L1 and L2 processing of Russian (L1) and English (L2) bilinguals, within the inferior frontal gyrus, L2 activated a larger cortical volume than L1 during lexical processing. This pattern (general overlap but specific distinction in L1 and L2 activation in left inferior temporal areas) has been replicated in more recent work (e.g., Nichols et al., 2021), providing congruent evidence with the hypotheses put forward in this paper that this distinct pattern of activation for L1 and L2 may be reflective of frequency-based cortical organization.

These findings collectively challenge the notion that language is the primary organizing principle in bilingual lexico-semantic processing. Instead, the behavioral pattern of deficits observed in this study, together with the location of the lesion, is consistent with the proposal that bilingual language organization operates on the same principles as monolingual organization (Blanco-Elorrieta & Caramazza, 2021); it just so happens that certain life experiences induce strong correlations between some of these variables (e.g., frequency) and language. Future research will have to specifically validate this hypothesis by looking at frequency-based cortical reorganization, which, if proven right, would support a language-agnostic model of cortical representation, governed by universal principles of frequency-based organization.

## ACKNOWLEDGMENTS

We sincerely thank the participants for their invaluable contribution to this study. We are also grateful to the Euskara Institutua (UPV/EHU) and, in particular, Josu Landa for providing lemma frequencies in Basque from the EHME database.

## FUNDING INFORMATION

Esti Blanco-Elorrieta, National Institute on Deafness and Other Communication Disorders (https://dx.doi.org/10.13039/100000055), Award ID: R00DC019973-03. Miren Arantzeta, Berrikuntza + Ikerketa + Osasuna Eusko Fundazioa (https://dx.doi.org/10.13039/501100012440), Award ID: IT1439/22-GIC21/132. Miren Arantzeta, Ministerio de Ciencia e Innovación (https://dx.doi.org/10.13039/501100004837), Award ID: RYC2021-033222-I. Miren Arantzeta, European Commission (https://dx.doi.org/10.13039/501100000780), Award ID: 10.13039/501100011033.

## AUTHOR CONTRIBUTIONS

**Esti Blanco-Elorrieta**: Conceptualization; Data curation; Funding acquisition; Visualization; Writing – original draft; Writing – review & editing. **Miren Arantzeta**: Conceptualization; Data curation; Formal analysis; Funding acquisition; Visualization; Writing – review & editing.

## DATA AND CODE AVAILABILITY STATEMENTS

All testing materials are available and can be found in the Supplementary Materials. All the data and code can be found in OSF: https://osf.io/xq6zy/.

## Supplementary Material









## TECHNICAL TERMS

Anomic aphasia: A language impairment where individuals struggle to retrieve words, despite understanding speech.
Cognates: Words in different languages that share similar sounds and meanings due to common origins.
Lesion mask: A digital outline used on MRI scans to identify and measure brain tissue damage.
Cohen’s kappa: A statistical measure evaluating agreement between two datasets beyond chance.
Error distribution analysis: Examining the patterns and frequency of mistakes to identify specific processing deficits.
Resting activation levels: Baseline neural activity in brain regions that influences processing efficiency without active tasks.
