# Supplementary material for: Basque-Spanish Bilingual Aphasia: A Case-Study to Reveal Frequency-Based, Language-Agnostic Lexical Organization in Bilinguals
Source: Neurobiol Lang (Camb). 2025 Jun 23;6:nol_a_00170. doi: 10.1162/nol_a_00170 (PMC12208705; doi:10.1162/nol_a_00170)
Supplement: Supplementary file 1 [file nol-6-1-170-s001.docx]

**Supplementary Materials:**

1. **Supplementary Material 1** contains the linguistic items used in the experiment for both Spanish and Basque, organized according to specific lexical characteristics. The items are classified by frequency and concreteness levels, enabling controlled comparisons between high- and low-frequency words, as well as high- and low-concreteness words. Each entry provides detailed information about the stimuli in both languages, including:

- Target Word (English Translation): The English equivalent of each target word.
- Item in Spanish: The target word in Spanish, including its orthographic length (number of letters) and log-transformed frequency value.
- Item in Basque: The target word in Basque, along with its orthographic length and log-transformed frequency value.
- Cognate Status: Indicates whether the Spanish and Basque items share a common form and meaning.
- Concreteness Rating: A numerical rating representing the concreteness level associated with each item.
- Condition: The lexical category of each item, based on combinations of frequency and concreteness (e.g., "Low Frequency – Low Concreteness").

1. **Supplementary Material 2** includes detailed information for the 180 target items, specifying their experimental condition, the corresponding definitions presented in Basque and Spanish, and an English translation of each definition.
2. **Supplementary Material 3** includes the summary of the participant’s performance on the Bilingual Aphasia Test (Basque-Spanish Adaptation) (Paradis, 1987; Erriondo, Álvarez, & Bidegain, 1989).
