# Supplementary material for: Basque-Spanish Bilingual Aphasia: A Case-Study to Reveal Frequency-Based, Language-Agnostic Lexical Organization in Bilinguals
Source: Neurobiol Lang (Camb). 2025 Jun 23;6:nol_a_00170. doi: 10.1162/nol_a_00170 (PMC12208705; doi:10.1162/nol_a_00170)
Supplement: Supplementary file 3 [file nol-6-1-170-s003.docx]

**Suplementary materials:**

The supplementary materials list all target words with their definitions in Basque, Spanish, and English. Each word is categorized by its experimental condition based on Frequency (High/Low) and Concreteness (High/Low), ensuring consistency across languages and transparency in the study design.

1. Target word: (EN) hair, (BA) ile, (SP) pelo - Condition: Concrete | Frequent

Definition (BA): Buru-azaletik ateratzen diren harizpi meheak, gehienetan kolore desberdinekoak, pertsonaren genetikaren arabera.

Definition (SP): Filamentos finos que salen del cuero cabelludo, generalmente de color variado, dependiendo de la genética de la persona.

"Fine filaments that grow from the scalp, typically varying in color depending on a person's genetics."

1. Target word: (EN) cat, (BA) katu, (SP) gato - Condition: Concrete | Frequent

Definition (BA): Etxeko ugaztun felinoa, arintasunagatik eta saguen ehiztaria izateagatik ezaguna.

Definition (SP): Mamífero felino doméstico que maulla, conocido por su agilidad y por ser un cazador de ratones.

"Domestic feline mammal that meows, known for its agility and as a mouse hunter."

1. Target word: (EN) eye, (BA) begi, (SP) ojo - Condition: Concrete | Frequent

Definition (BA): Aurpegian dauden zentzumen-organoak, argia eta formak hautematen laguntzen digutenak, kolore desberdinetako irisekin.

Definition (SP): Órganos sensoriales en la cara que nos permiten percibir la luz y las formas, con iris de colores variados.

"Sensory organs on the face that allow us to perceive light and shapes, with irises in various colors."

1. Target word: (EN) window, (BA) leiho, (SP) ventana - Condition: Concrete | Frequent

Definition (BA): Eraikin bateko hormetako irekidura, zeinek argia eta airea sartzeko aukera ematen duen, normalean kristalezkoak izanik.

Definition (SP): Abertura en una pared de un edificio que permite la entrada de luz y aire, comúnmente de cristal.

"Opening in a building wall that allows the entry of light and air, commonly made of glass."

1. Target word: (EN) ship, (BA) itsasontzi, (SP) barco - Condition: Concrete | Frequent

Definition (BA): Ur gaineko garraiobidea, antzinatik erabilia, pirateriaren aroa barne.

Definition (SP): Medio de transporte sobre agua, usado desde tiempos antiguos, incluida la era de la piratería.

"Water transport vehicle, used since ancient times, including the era of piracy."

1. Target word: (EN) bed, (BA) ohe, (SP) cama - Condition: Concrete | Frequent

Definition (BA): Lotarako altzaria, hankak eta koltxoi erosoa dituena, funtsezkoa edozein etxetan.

Definition (SP): Mueble para dormir que consiste en una base con patas y un colchón cómodo, esencial en cualquier hogar.

"Furniture for sleeping that consists of a base with legs and a comfortable mattress, essential in any home."

1. Target word: (EN) arm, (BA) beso, (SP) brazo - Condition: Concrete | Frequent

Definition (BA): Giza gorputzaren goiko gorputz-adarra, artikulatua eta malgua, sorbaldatik eskura luzatzen dena.

Definition (SP): Extremidad superior del cuerpo humano, articulado y flexible, se extiende desde el hombro hasta la mano.

"Upper limb of the human body, articulated and flexible, extending from the shoulder to the hand. "

1. Target word: (EN) tree, (BA) zuhaitz, (SP) árbol - Condition: Concrete | Frequent

Definition (BA): Enbor zurkara, adarrak eta hostoak dituen landare handia, urte askoan bizi daitekeena, hala nola haritza edo pinua.

Definition (SP): Planta grande que tiene un tronco leñoso, ramas y hojas, y puede vivir muchos años, como el roble o el pino.

"Large plant with a woody trunk, branches, and leaves, capable of living many years, like the oak or pine."

1. Target word: (EN) box, (BA) kutxa, (SP) caja - Condition: Concrete | Frequent

Definition (BA): Edukiontzi angeluzuzena, estalkiduna, hainbat objektu gordetzeko edo garraiatzeko erabiltzen dena, askotan kartoi edo plastikozkoa.

Definition (SP): Contenedor rectangular con tapa, utilizado para almacenar o transportar diversos objetos, a menudo de cartón o plástico.

"Rectangular container with a lid, used to store or transport various objects, often made of cardboard or plastic."

1. Target word: (EN) star, (BA) izar, (SP) estrella - Condition: Concrete | Frequent

Definition (BA): Plasmaz osatutako zeruko-gorputza, gauean distira egiten duena, barruan dituen erreakzio nuklearren ondorioz.

Definition (SP): Cuerpo celeste compuesto de plasma que brilla en la noche debido a las reacciones nucleares en su interior.

"Celestial body composed of plasma that shines at night due to nuclear reactions within it."

1. Target word: (EN) tower, (BA) dorre, (SP) torre - Condition: Concrete | Frequent

Definition (BA): Egitura arkitektoniko altu eta bertikala, gune handiago baten zati izan daitekeena, hala nola gaztelu edo gotorleku baten zati.

Definition (SP): Estructura arquitectónica elevada y vertical que puede ser parte de un complejo mayor como un castillo o una fortaleza.

"Elevated and vertical architectural structure that can be part of a larger complex, such as a castle or fortress."

1. Target word: (EN) road, (BA) errepide, (SP) carretera - Condition: Concrete | Frequent

Definition (BA): Ibilgailuak eta pertsonak leku batetik bestera eramateko bide pabimentatua.

Definition (SP): Camino pavimentado que facilita el transporte terrestre de vehículos y personas de un lugar a otro.

"Paved road that facilitates the land transport of vehicles and people from one place to another."

1. Target word: (EN) animal, (BA) animalia, (SP) animal - Condition: Concrete | Frequent

Definition (BA): Basatia izan daitekeen biziduna, zoologikoetan bizi daitekeena, edota etxeetan maskota gisa zaindu daitekeena.

Definition (SP): Ser vivo que puede ser salvaje, habitar en zoológicos, o ser cuidado en hogares como mascota.

"Living being that can be wild, inhabit zoos, or be cared for in homes as a pet."

1. Target word: (EN) moon, (BA) ilargi, (SP) luna - Condition: Concrete | Frequent

Definition (BA): Gorputz zerutiarra, Lurraren inguruan biraka dabilena eta gauero fasez aldatzen dena.

Definition (SP): Cuerpo celeste que gira alrededor de la Tierra y cambia de fase visiblemente cada noche.

"Heavenly body that orbits around the Earth and visibly changes phase each night."

1. Target word: (EN) stone, (BA) harri, (SP) piedra - Condition: Concrete | Frequent

Definition (BA): Askotariko gogortasuna duten mineralez osatutako objektu solido eta naturala, hainbat forma eta kolore izan ditzakeena.

Definition (SP): Objeto sólido y natural compuesto de minerales, de dureza variada, y que puede tener diferentes formas y colores.

"Solid, natural object composed of minerals, with varying hardness, and which can have different shapes and colors."

1. Target word: (EN) horse, (BA) zaldi, (SP) caballo - Condition: Concrete | Frequent

Definition (BA): Lau hankako ugaztun belarjalea, gurdiak muntatu edo tiratzeko erabiltzen dena eta hipiketan entrenatu daitekeena.

Definition (SP): Mamífero herbívoro de cuatro patas, usado para montar o tirar de carruajes, y entrenado en hípicas.

"Four-legged herbivorous mammal, used for riding or pulling carriages, and trained in equestrian activities."

1. Target word: (EN) mouth, (BA) aho, (SP) boca - Condition: Concrete | Frequent

Definition (BA): Giza gorputzaren zulo edo barrunbea, elikagaiak hartu eta komunikaziorako soinuak artikulatzen dituena.

Definition (SP): Cavidad oral del cuerpo humano por la que se ingieren alimentos y se articulan sonidos para la comunicación.

"Oral cavity of the human body through which food is ingested and sounds are articulated for communication."

1. Target word: (EN) child, (BA) ume, (SP) niño - Condition: Concrete | Frequent

Definition (BA): Jolasaren bidez ezagutzak eta trebetasunak bereganatzen dituen gizaki txikia.

Definition (SP): Individuo infantil de especie humana que adquiere conocimientos y habilidades mediante el juego.

"Infant individual of the human species who acquires knowledge and skills through play."

1. Target word: (EN) table, (BA) mahai, (SP) mesa - Condition: Concrete | Frequent

Definition (BA): Hankez eutsitako gainazal lau batez osatutako altzariak, jateko edo lan egiteko erabiltzen direnak.

Definition (SP): Mobiliario compuesto por una superficie plana sostenida por patas, utilizado en actividades como comer o trabajar.

"Furniture composed of a flat surface supported by legs, used for activities such as eating or working."

1. Target word: (EN) radio, (BA) irrati, (SP) radio - Condition: Concrete | Frequent

Definition (BA): Uhin elektromagnetikoak jasotzen dituen aparatua, musika eta albisteak bezalako soinu-transmisioak entzuteko aukera ematen duena.

Definition (SP): Aparato receptor de ondas electromagnéticas que permite escuchar transmisiones sonoras como música y noticias.

"Device that receives electromagnetic waves, allowing the listening of sound transmissions like music and news."

1. Target word: (EN) blood, (BA) odol, (SP) sangre - Condition: Concrete | Frequent

Definition (BA): Kolore gorriko ehun konektibo likidoa, sistema baskularrean oxigenoa eta mantenugaiak garraiatzen dituena.

Definition (SP): Tejido conectivo líquido de color rojo que circula por el sistema vascular transportando oxígeno y nutrientes.

"Red-colored liquid connective tissue that circulates through the vascular system, transporting oxygen and nutrients."

1. Target word: (EN) cross, (BA) gurutze, (SP) cruz - Condition: Concrete | Frequent

Definition (BA): Kristautasunari lotutako sinbolo erlijiosoa, Jesukristoren heriotza irudikatzen duena.

Definition (SP): Símbolo religioso asociado al cristianismo, que representa el instrumento de la muerte de Jesucristo.

"Religious symbol associated with Christianity, representing the instrument of Jesus Christ's death."

1. Target word: (EN) foot, (BA) oin, (SP) pie - Condition: Concrete | Frequent

Definition (BA): Gorputzaren pisua jasan eta mugimendua errazten duen hankaren azken zatia, zeinetan ohinetakoak jasten ditugun normalean.

Definition (SP): Parte final de la pierna que soporta el peso corporal y facilita el movimiento, típicamente calzada.

"Final part of the leg that supports body weight and facilitates movement, typically covered by footwear."

1. Target word: (EN) fountain, (BA) iturri, (SP) fuente - Condition: Concrete | Frequent

Definition (BA): Ura isurtzen duen egitura, sarritan leku publikoetan aurkitzen dena, hala nola parke edo plazetan.

Definition (SP): Estructura que emite agua, frecuentemente encontrada en lugares públicos como parques o plazas.

"Structure that releases water, commonly found in public places like parks or squares."

1. Target word: (EN) port, (BA) portu, (SP) puerto - Condition: Concrete | Frequent

Definition (BA): Itsasertzeko tokia ontziak iritsi eta ateratzeko eta salgaiak mugitzeko egokitua.

Definition (SP): Zona costera acondicionada para la llegada y salida de embarcaciones, así como para el movimiento de mercancías.

"Coastal area equipped for the arrival and departure of vessels, as well as for the movement of goods."

1. Target word: (EN) face, (BA) aurpegia, (SP) cara - Condition: Concrete | Frequent

Definition (BA): Giza buruaren aurrealdea, begiak, sudurra eta ahoa bezalako elementuak dituena eta emozioak adierazten dituena.

Definition (SP): Parte frontal de la cabeza humana que incluye rasgos como los ojos, la nariz y la boca, expresando emociones.

"Front part of the human head that includes features like the eyes, nose, and mouth, expressing emotions."

1. Target word: (EN) color, (BA) kolore, (SP) color - Condition: Concrete | Frequent

Definition (BA): Objektuen gainazalean hauteman daitekeen propietatea, eguzkiaren argia edo argi artifiziala islatzeko moduaren ondorioz.

Definition (SP): Propiedad perceptible en la superficie de los objetos debido a la manera en que reflejan la luz solar o artificial.

"Perceptible property on the surface of objects due to the way they reflect sunlight or artificial light."

1. Target word: (EN) cinema, (BA) zinema, (SP) cine - Condition: Concrete | Frequent

Definition (BA): Entretenimendurako tokia, non talde-gozamenerako filmak eskaintzen diren pantaila handi batean.

Definition (SP): Espacio de entretenimiento donde se proyectan películas en una pantalla grande para el disfrute colectivo.

"Entertainment space where movies are projected on a large screen for collective enjoyment."

1. Target word: (EN) television, (BA) telebista, (SP) televisión - Condition: Concrete | Frequent

Definition (BA): Ikus-entzunezko programak eta edukiak aurkezteko seinaleak jasotzen dituen gailua, entretenimendu modernoaren funtsezko elementua.

Definition (SP): Dispositivo que recibe señales para presentar programas y contenido audiovisual, parte esencial del entretenimiento moderno.

"Device that receives signals to present programs and audiovisual content, an essential part of modern entertainment."

1. Target word: (EN) school, (BA) eskola, (SP) escuela - Condition: Concrete | Frequent

Definition (BA): Irakaskuntzarako lekua, non haur eta nerabeek hezkuntza formala jasotzen duten hainbat gaitan.

Definition (SP): Establecimiento dedicado a la enseñanza, donde niños y adolescentes reciben educación formal en diversas materias.

"Establishment dedicated to teaching, where children and adolescents receive formal education in various subjects."

1. Target word: (EN) street, (BA) kale, (SP) calle - Condition: Concrete | Frequent

Definition (BA): Ibilgailuak eta pertsonak ibiltzen diren eremu publikoa, hiri orientaziorako eta posta helbiderako berariazko izena jasotzen duena.

Definition (SP): Vía pública por donde circulan vehículos y personas, con nomenclatura para la orientación urbana y la dirección postal.

"Public road where vehicles and people circulate, with names for urban orientation and postal addressing."

1. Target word: (EN) sun, (BA) eguzki, (SP) sol - Condition: Concrete | Frequent

Definition (BA): Gure eguzki-sistemako izar nagusia, Lurreko bizitzarako funtsezkoak diren argia eta beroa ematen dituena.

Definition (SP): Estrella central de nuestro sistema solar que proporciona luz y calor esenciales para la vida en la tierra.

"Central star of our solar system that provides essential light and heat for life on Earth."

1. Target word: (EN) gold, (BA) urre, (SP) oro - Condition: Concrete | Frequent

Definition (BA): Metal noble eta preziatua, kolore hori bereizgarrikoa, bitxigintzan asko baloratua, baita balio ekonomikoko erreserba gisa.

Definition (SP): Metal noble y precioso, de color amarillo característico, muy valorado en joyería y como reserva de valor económico.

"Noble and precious metal with a characteristic yellow color, highly valued in jewelry and as an economic store of value."

1. Target word: (EN) heart, (BA) bihotz, (SP) corazón - Condition: Concrete | Frequent

Definition (BA): Zirkulazio-sistemaren bidez odola ponpatzen duen muskulu-organoa, giza funtzio biologikorako ezinbestekoa.

Definition (SP): Órgano muscular que bombea la sangre a través del sistema circulatorio, vital para la función biológica humana.

"Muscular organ that pumps blood through the circulatory system, essential for human biological function."

1. Target word: (EN) island, (BA) uharte / irla, (SP) isla - Condition: Concrete | Frequent

Definition (BA): Urez guztiz inguratutako lur-zatia, sarritan jendea bizi dena eta berezko ekosistemak dituena.

Definition (SP): Porción de tierra rodeada completamente por agua, a menudo habitada y con ecosistemas propios.

"Portion of land completely surrounded by water, often inhabited and with its own ecosystems."

1. Target word: (EN) door, (BA) ate, (SP) puerta - Condition: Concrete | Frequent

Definition (BA): Eraikin edo geletako sarrerako elementu mugikorra, itxita dagoenean sarbidea edo pribatutasuna ahalbidetzen duena.

Definition (SP): Elemento móvil en la entrada de edificios o habitaciones que permite el acceso o la privacidad cuando está cerrada.

"Movable element at the entrance of buildings or rooms that allows access or privacy when closed."

1. Target word: (EN) money, (BA) diru, (SP) dinero - Condition: Concrete | Frequent

Definition (BA): Ondasunen eta zerbitzuen erosketan eta salmentan transakzio ekonomikoetarako erabiltzen den elementua.

Definition (SP): Elemento utilizado para transacciones económicas en la compra y venta de bienes y servicios.

"Element used for economic transactions in the buying and selling of goods and services."

1. Target word: (EN) book, (BA) liburu, (SP) libro - Condition: Concrete | Frequent

Definition (BA): Paperezko orri inprimatu eta koadernatuen multzoa, informazio edo literatura testuak dituztenak.

Definition (SP): Conjunto de hojas de papel impresas y encuadernadas que contienen textos informativos o literarios.

"A set of printed and bound paper sheets containing informational or literary texts."

1. Target word: (EN) church, (BA) eliza, (SP) iglesia - Condition: Concrete | Frequent

Definition (BA): Kristau-fedearen kultua eta bilerak egiteko eraikina, sarritan arkitektura bereizgarriarekin eta aldarearekin.

Definition (SP): Edificio dedicado al culto y reuniones de la fe cristiana, a menudo con arquitectura distintiva y un altar.

"Building dedicated to worship and Christian faith gatherings, often featuring distinctive architecture and an altar."

1. Target word: (EN) head, (BA) buru, (SP) cabeza - Condition: Concrete | Frequent

Definition (BA): Garuna eta beste zentzumen-organo batzuk, hala nola begiak eta entzumena, dituen gorputzaren goiko aldea.

Definition (SP): Parte superior del cuerpo que alberga el cerebro y otros órganos sensoriales, como los ojos y el oído.

"Upper part of the body that houses the brain and other sensory organs, such as the eyes and ears."

1. Target word: (EN) body, (BA) gorputz, (SP) cuerpo - Condition: Concrete | Frequent

Definition (BA): Organismo bizi baten egitura fisikoa eta funtzionala osatzen duten sistema eta organoen multzoa.

Definition (SP): Conjunto de sistemas y órganos que constituyen la estructura física y funcional de un organismo vivo.

"Set of systems and organs that make up the physical and functional structure of a living organism."

1. Target word: (EN) water, (BA) ur, (SP) agua - Condition: Concrete | Frequent

Definition (BA): Bizitzeko funtsezko baliabide naturala, hainbat egoeratan naturan dagoen hidrogenoz eta oxigenoz osatua.

Definition (SP): Recurso natural esencial para la vida, compuesto por hidrógeno y oxígeno, presente en la naturaleza en varios estados.

"Essential natural resource for life, composed of hydrogen and oxygen, present in nature in various states."

1. Target word: (EN) king, (BA) errege, (SP) rey - Condition: Concrete | Frequent

Definition (BA): Gobernu hereditario baten monarka eta estatuburua, sarritan koroa batekin sinbolizatzen dena.

Definition (SP): Monarca y jefe de estado de una forma de gobierno hereditaria, a menudo simbolizado por una corona.

"Monarch and head of state in a hereditary form of government, often symbolized by a crown."

1. Target word: (EN) house, (BA) etxe, (SP) casa - Condition: Concrete | Frequent

Definition (BA): Pertsonentzako bizileku gisa diseinatutako eraikina, aterpea eta erosotasuna ematen dituena; normalean, hainbat logela eta oinarrizko zerbitzu izaten ditu.

Definition (SP): Construcción diseñada como lugar de residencia para las personas, que proporciona refugio y confort, habitualmente incluye varias habitaciones y servicios básicos.

"Structure designed as a residence for people, providing shelter and comfort, typically includes several rooms and basic amenities."

1. Target word: (EN) man, (BA) gizon, (SP) hombre - Condition: Concrete | Frequent

Definition (BA): Giza espezieko pertsona heldu, ezaugarri biologiko eta fisiologiko maskulinoak dituena.

Definition (SP): Individuo adulto de género masculino con rasgos biológicos y fisiológicos únicos.

"Adult individual of the male gender with unique biological and physiological traits."

1. Target word: (EN) heel, (BA) takoi, (SP) tacon - Condition: Concrete | Infrequent

Definition (BA): Zapata batzuk atzeko aldean duten gehigarria, erabiltzen duenaren altuera handitzeko, itxura estilizatua emanez.

Definition (SP): Elevación en la parte trasera del zapato que realza la altura de quien lo usa, aportando un aspecto estilizado.

"Elevation at the back of the shoe that enhances the height of the wearer, adding a stylized appearance."

1. Target word: (EN) coffin, (BA) hilkutxa, (SP) ataud - Condition: Concrete | Infrequent

Definition (BA): Ontzi angeluzuzena, gehienetan egurrezkoa, giza gorputza gordetzeko eta hileta batean lur azpian uzteko diseinatua.

Definition (SP): Recipiente rectangular, usualmente de madera, diseñado para albergar un cuerpo humano y ser depositado en la tierra durante un funeral.

"Rectangular container, usually made of wood, designed to hold a human body and be laid to rest in the ground during a funeral."

1. Target word: (EN) driving school, (BA) autoeskola, (SP) autoescuela - Condition: Concrete | Infrequent

Definition (BA): Ikasleei ibilgailuak gidatzeko trebetasun praktiko eta teorikoak irakasten dizkien tokia.

Definition (SP): Institución educativa donde los instructores enseñan a los estudiantes las habilidades prácticas y teóricas para conducir vehículos.

"Educational institution where instructors teach students the practical and theoretical skills needed to drive vehicles."

1. Target word: (EN) cheek, (BA) masail, (SP) mejilla - Condition: Concrete | Infrequent

Definition (BA): Giza aurpegiaren gunea emozioaren edo beroaren eraginez gorritu daitekeena.

Definition (SP): Zona del rostro humano que puede enrojecerse por efecto de la emoción o el calor.

"Area of the human face that can redden due to emotion or heat."

1. Target word: (EN) salmon, (BA) izokin, (SP) salmon - Condition: Concrete | Infrequent

Definition (BA): Arrain arrosa, gastronomian preziatua, maiz ketua eta omeg-3 ugari duena.

Definition (SP): Pescado de tonalidad rosada, apreciado en gastronomía, frecuentemente consumido ahumado y rico en omega-3.

"Fish with a pinkish hue, valued in gastronomy, often consumed smoked and rich in omega-3."

1. Target word: (EN) corn, (BA) arto, (SP) maiz - Condition: Concrete | Infrequent

Definition (BA): Ale horiak, gastronomian erabiliak, krispetak, zerealezko tortillak eta beste platertxo batzuk prestatzeko erabiltzen direnak.

Definition (SP): Cultivo de granos amarillos, versátil en gastronomía, para preparar desde palomitas hasta tortillas de cereales y otros platos.

"Cultivation of yellow grains, versatile in gastronomy, used to prepare everything from popcorn to cereal tortillas and other dishes."

1. Target word: (EN) bulb, (BA) bonbilla, (SP) bombilla - Condition: Concrete | Infrequent

Definition (BA): Makats itxurako argi-iturri elektrikoa, espazioak argiztatzeko lanparetan jartzen dena.

Definition (SP): Fuente de iluminación eléctrica con forma de pera que se enrosca en lámparas para iluminar espacios.

"Electric light source in the shape of a pear that screws into lamps to illuminate spaces."

1. Target word: (EN) tractor, (BA) traktore, (SP) tractor - Condition: Concrete | Infrequent

Definition (BA): Nekazaritzako ibilgailu astuna, nekazaritza modernoan ezinbestekoa zelaia goldatu eta kargak eramateko.

Definition (SP): Vehículo agrícola pesado, indispensable en la agricultura moderna para arar y trasladar cargas en el campo.

"Heavy agricultural vehicle, essential in modern farming for plowing and transporting loads in the field."

1. Target word: (EN) puppet, (BA) txotxongilo, (SP) marioneta - Condition: Concrete | Infrequent

Definition (BA): Hari edo barillez maneiatutako panpin artikulatua, entretenimenduko eta antzerkiko ikuskizunetan erabilia.

Definition (SP): Muñeco articulado manejado por hilos o varillas, utilizado en espectáculos de entretenimiento y teatro.

"Articulated doll controlled by strings or rods, used in entertainment shows and theater."

1. Target word: (EN) flea, (BA) arkakuso, (SP) pulga - Condition: Concrete | Infrequent

Definition (BA): Parasito ñimiño eta saltarina, txakurrak edo katuak bezalako maskotetan aurkitzen dira eta azkura eragiten dute.

Definition (SP): Parásito diminuto y saltarín, comúnmente hallado en mascotas como perros y gatos, causante de picazón.

"Tiny, jumping parasite commonly found on pets like dogs and cats, causing itching."

1. Target word: (EN) camel, (BA) gamelu, (SP) camello - Condition: Concrete | Infrequent

Definition (BA): Basamortuko ugaztuna, koipea metatzen duten jorobengatik ezaguna, klima lehorretara egokitua.

Definition (SP): Mamífero del desierto conocido por sus jorobas que almacenan grasa, adaptado a climas áridos.

"Desert mammal known for its humps that store fat, adapted to arid climates."

1. Target word: (EN) owl, (BA) hontz, (SP) búho - Condition: Concrete | Infrequent

Definition (BA): Gaueko hegaztia, isila, begi handiekin eta ikusmen akutuarekin, jakinduriari lotua.

Definition (SP): Ave nocturna de presencia silenciosa con ojos grandes y visión aguda, asociada a la sabiduría.

"Nocturnal bird with a silent presence, large eyes, and sharp vision, often associated with wisdom."

1. Target word: (EN) chicken run, (BA) oilotegi, (SP) gallinero - Condition: Concrete | Infrequent

Definition (BA): Oiloak zaindu eta hazteko gunea, haiek babestuz eta arrautzak jartzeko lekua emanez.

Definition (SP): Espacio destinado al alojamiento y cría de gallinas, protegiéndolas y proporcionando un lugar para poner huevos.

"Space designated for the housing and breeding of hens, protecting them and providing a place to lay eggs."

1. Target word: (EN) cereal, (BA) zereal, (SP) cereal - Condition: Concrete | Infrequent

Definition (BA): Gosarian erabilitako ale begetala, normalean birrindua edo puztua, eta esne edo jogurtarekin zerbitzatua.

Definition (SP): Grano vegetal utilizado en el desayuno, comúnmente triturado o inflado y servido con leche o yogur.

"Vegetable grain used for breakfast, commonly crushed or puffed and served with milk or yogurt."

1. Target word: (EN) furniture, (BA) altzari, (SP) mueble - Condition: Concrete | Infrequent

Definition (BA): Etxe eta bulegoetan dauden dekorazio eta erabilera artikuluak, hala nola aulkiak, mahaiak eta apalategiak.

Definition (SP): Artículos de decoración y utilidad en hogares y oficinas, como sillas, mesas y estanterías.

"Items of decoration and utility for homes and offices, such as chairs, tables, and shelves."

1. Target word: (EN) palm tree, (BA) palmondo, (SP) palmera - Condition: Concrete | Infrequent

Definition (BA): Landare tropikala, enbor luzekoa eta haizemaile-hostoak dituena, datil elikagarrien iturria.

Definition (SP): Planta tropical característica por su tronco largo y hojas en abanico, fuente de dátiles nutritivos.

"Tropical plant characterized by its tall trunk and fan-shaped leaves, a source of nutritious dates."

1. Target word: (EN) bacon, (BA) hirugihar, (SP) bacon - Condition: Concrete | Infrequent

Definition (BA): Txerri-tira ondu eta ketuak, osagai klasikoa gosarietan, batez ere Estatu Batuetako sukaldaritzan.

Definition (SP): Tiras de cerdo curado y ahumado, ingrediente clásico en desayunos, especialmente en la cocina estadounidense.

"Strips of cured and smoked pork, a classic ingredient in breakfasts, especially in American cuisine."

1. Target word: (EN) rag, (BA) trapu, (SP) trapo - Condition: Concrete | Infrequent

Definition (BA): Oihal xurgatzailea, etxea garbitzeko eta mantentzeko lanetan erabiltzen dena.

Definition (SP): Pieza de tela absorbente utilizada en tareas de limpieza y mantenimiento del hogar.

"Absorbent piece of fabric used for cleaning tasks and home maintenance."

1. Target word: (EN) shark, (BA) marrazo, (SP) tiburón - Condition: Concrete | Infrequent

Definition (BA): Tamainagatik eta hortz zorrotzengatik beldurgarria den eskualoa, itsas ekosistemetako harrapari garrantzitsua.

Definition (SP): Escualo temido por su tamaño y dientes afilados, importante depredador en ecosistemas marinos.

"Shark feared for its size and sharp teeth, an important predator in marine ecosystems."

1. Target word: (EN) dungeon, (BA) ziega, (SP) calabozo/mazmorra - Condition: Concrete | Infrequent

Definition (BA): Erdi Aroko gazteluen azpian egon ohi den espazioa, askotan iluna eta hezea.

Definition (SP): Espacio de reclusión antiguo, a menudo oscuro y húmedo, típicamente bajo castillos medievales.

"Ancient confinement space, often dark and damp, typically beneath medieval castles."

1. Target word: (EN) costume, (BA) mozorro, (SP) disfraz - Condition: Concrete | Infrequent

Definition (BA): Jaietan eta kultur ospakizunetan, hala nola Inauterietan, erabili ohi den pertsonaia edo gai jakin bat irudikatzeko diseinatutako jantzia.

Definition (SP): Prenda diseñada para encarnar un personaje o tema específico, comúnmente usada en eventos festivos y celebraciones culturales como los Carnavales.

"Garment designed to embody a specific character or theme, commonly worn at festive events and cultural celebrations like Carnivals."

1. Target word: (EN) deer, (BA) orein, (SP) ciervo - Condition: Concrete | Infrequent

Definition (BA): Basoko belarjale dotorea, Bambi film animatuan bere adar eta irudi ikonikoegatik ezaguna.

Definition (SP): Herbívoro elegante del bosque, conocido por sus astas y la figura icónica en la película animada Bambi.

"Elegant forest herbivore, known for its antlers and iconic figure in the animated movie *Bambi*."

1. Target word: (EN) childbirth, (BA) erditze, (SP) parto - Condition: Concrete | Infrequent

Definition (BA): Gertaera biologiko naturala, haur baten jaiotzari leku ematen diona 9 hilabeteko ernaldiaren ostean.

Definition (SP): Evento biológico natural que da lugar al nacimiento de un bebé despues de 9 meses de gestación.

"Natural biological event that results in the birth of a baby after 9 months of gestation."

1. Target word: (EN) taste, (BA) zapore, (SP) sabor - Condition: Concrete | Infrequent

Definition (BA): Aho-sapaian jasotzen diren askotariko dastamen-pertzepzioak, besteak beste, gozoa, gazia, mikatza, azidoa eta umamia

Definition (SP): Variedad de percepciones gustativas que incluyen lo dulce, salado, amargo, ácido y umami detectadas por el paladar.

"Variety of taste perceptions that include sweet, salty, bitter, sour, and umami, detected by the palate."

1. Target word: (EN) bee, (BA) erle, (SP) abeja - Condition: Concrete | Infrequent

Definition (BA): Eztia sortzen duen intsektu hegalaria, gorputz marraduna, polinizaziorako eta biodibertsitaterako ezinbestekoa.

Definition (SP): Insecto volador productor de miel, con cuerpo rayado, crucial para la polinización y biodiversidad.

"Flying insect that produces honey, with a striped body, crucial for pollination and biodiversity."

1. Target word: (EN) turtle, (BA) dortoka, (SP) tortuga - Condition: Concrete | Infrequent

Definition (BA): Mugimendu moteleko narrasti urtarra edo lurrekoa, gorputza babesten duen oskol gogorrarekin.

Definition (SP): Reptil acuático o terrestre de movimiento lento con un caparazón duro que protege su cuerpo.

"Aquatic or terrestrial reptile with slow movement and a hard shell that protects its body."

1. Target word: (EN) tooth, (BA) hortz, (SP) diente - Condition: Concrete | Infrequent

Definition (BA): Ahoko hezur-egitura, elikagaiak murtxikatzeko funtsezkoa, irribarre egitean ikusgarria.

Definition (SP): Estructura ósea en la boca, esencial para masticar alimentos, visible al sonreír.

"Bone structure in the mouth, essential for chewing food, visible when smiling."

1. Target word: (EN) thorn, (BA) arantza, (SP) espina - Condition: Concrete | Infrequent

Definition (BA): Landare baten apendize zorrotza, animalia belarjaleen kontra egiten duena, arrosetan eta beste zuhaixka batzuetan ohikoa dena.

Definition (SP): Apéndice afilado que se encuentra en plantas que se defienden contra animales herbívoros; típico en rosas y otros arbustos.

"Sharp appendage found in plants that defend against herbivorous animals; typical in roses and other shrubs."

1. Target word: (EN) ash, (BA) errauts, (SP) ceniza - Condition: Concrete | Infrequent

Definition (BA): Materialen errekuntza osoaren ondorioz sortutako hauts-hondakinak, gehienetan gris kolorekoak.

Definition (SP): Residuo en polvo resultante de la combustión completa de materiales, generalmente de color gris.

"Powdery residue resulting from the complete combustion of materials, usually gray in color."

1. Target word: (EN) wrist, (BA) eskumutur, (SP) muñeca - Condition: Concrete | Infrequent

Definition (BA): Eskua besaurrearekin lotzen duen giza besoaren segmentu artikulatua, non erloju gisako apaingarriak jartzen diren.

Definition (SP): Segmento articulado del brazo humano que conecta la mano con el antebrazo, donde se colocan adornos como relojes.

"Articulated segment of the human arm that connects the hand to the forearm, where accessories like watches are worn."

1. Target word: (EN) flute, (BA) txirula, (SP) flauta - Condition: Concrete | Infrequent

Definition (BA): Horizontalean eusten den haize-instrumentu melodikoa, zuloak hatzekin itxi eta putz egitean ukitzen dena.

Definition (SP): Instrumento melódico de viento que se sostiene horizontalmente, tocado al soplar y cerrar sus orificios con los dedos.

"Melodic wind instrument held horizontally, played by blowing and covering its holes with the fingers."

1. Target word: (EN) button, (BA) botoi, (SP) botón - Condition: Concrete | Infrequent

Definition (BA): Arropa lotzeko edo gailu elektronikoak eta makinak erabiltzeko erabiltzen den pieza trinkoa eta zirkularra.

Definition (SP): Pieza compacta y circular utilizada para abrochar la ropa o como interfaz para operar con dispositivos electrónicos y máquinas.

"Compact, circular piece used to fasten clothing or as an interface to operate electronic devices and machines."

1. Target word: (EN) brick, (BA) adreilu, (SP) ladrillo - Condition: Concrete | Infrequent

Definition (BA): Funtsezko elementua eraikuntzan, iraunkorra eta normalean gorrixka, egitura egonkorrak sortzeko erabiltzen dena, hala nola hormak eta eraikinak.

Definition (SP): Elemento fundamental en construcción, durable y típicamente rojizo, utilizado en la creación de estructuras estables como muros y edificios.

"Essential construction element, durable and typically reddish, used in creating stable structures such as walls and buildings."

1. Target word: (EN) comics, (BA) komiki, (SP) comics - Condition: Concrete | Infrequent

Definition (BA): Istorioak ilustrazioen eta elkarrizketen bidez kontatzen dituzten argitalpen grafiko narratiboak, hala nola Batman edo Spiderman.

Definition (SP): Publicaciones gráficas narrativas que cuentan historias a través de ilustraciones y diálogos, como Batman o Spiderman.

"Graphic narrative publications that tell stories through illustrations and dialogue, like Batman or Spider-Man."

1. Target word: (EN) frog, (BA) igel, (SP) rana - Condition: Concrete | Infrequent

Definition (BA): Kolore berdeko anfibio jauzilaria, kantu bereizgarriagatik eta hezeguneetan duen habitatagatik ezaguna.

Definition (SP): Anfibio saltador de color verde, conocido por su canto distintivo y su hábitat en zonas húmedas.

"Green jumping amphibian, known for its distinctive croak and its habitat in wetland areas."

1. Target word: (EN) mill, (BA) errota, (SP) molino - Condition: Concrete | Infrequent

Definition (BA): Aleak ehotzeko egitura, energia eoliko edo hidraulikoak bultzatua, biratzen diren hegalekin.

Definition (SP): Estructura para moler granos, impulsada por energía eólica o hidráulica, con aspas que giran.

"Structure for grinding grains, powered by wind or water energy, with rotating blades."

1. Target word: (EN) circus, (BA) zirku, (SP) circo - Condition: Concrete | Infrequent

Definition (BA): Pailazoen, akrobaten, animalien hezitzaileen eta malabaristen emanaldiekin entretenitzeko tokia edo gunea, normalean karpa batean instalatua.

Definition (SP): Lugar o espacio de entretenimiento con actuaciones de payasos, acróbatas, domadores de animales y malabaristas, usualmente instalado en una carpa.

"Place or space for entertainment featuring performances by clowns, acrobats, animal trainers, and jugglers, usually set up in a tent."

1. Target word: (EN) hive, (BA) erlauntz, (SP) colmena - Condition: Concrete | Infrequent

Definition (BA): Erleek eztia gorde eta bizitzeko sortutako argizari-egitura, ezinbestekoa polinizatzeko laboreetan.

Definition (SP): Estructura de cera creada por abejas para vivir y almacenar miel, vital para la polinización de cultivos.

"Wax structure created by bees to live in and store honey, vital for crop pollination."

1. Target word: (EN) dampness, (BA) hezetasun, (SP) humedad - Condition: Concrete | Infrequent

Definition (BA): Aireko ur-lurrunarek neurketa zeinek kliman eta inguruko erosotasun-sentsazioan eragiten duen.

Definition (SP): Medida de vapor de agua en el aire que influye en el clima y la sensación de confort ambiental.

"Measure of water vapor in the air that influences the climate and the feeling of environmental comfort."

1. Target word: (EN) lamb, (BA) arkume, (SP) cordero - Condition: Concrete | Infrequent

Definition (BA): Sarritan haragi samur eta artileagatik hazten den ardi gaztea, hainbat kultura gastronomikotan kontsumitua.

Definition (SP): Joven ovino frecuentemente criado por su carne tierna y lana, consumido en diversas culturas gastronómicas.

"Young sheep commonly raised for its tender meat and wool, consumed in various gastronomic cultures."

1. Target word: (EN) straw, (BA) lasto, (SP) paja - Condition: Concrete | Infrequent

Definition (BA): Animalien elikaduran edo ohantzean erabiltzen diren belar zurtoin lehorrak, baita artisautzan ere.

Definition (SP): Tallos secos de hierba usados para la alimentación animal o como lecho, también en artesanía.

"Dry grass stems used for animal feed or bedding, also in crafts."

1. Target word: (EN) dragon, (BA) dragoi, (SP) dragón - Condition: Concrete | Infrequent

Definition (BA): Hegoak dituen eta sua arnasten edo kanporatzen duen animalia edo ipuineko izaki mitologikoa, kontakizun fantastikoetan agertzen dena.

Definition (SP): Animal o criatura mitológica de cuento que tiene alas y exhala o respira fuego, presente en relatos fantásticos.

"Mythological animal or creature from tales that has wings and exhales or breathes fire, present in fantastic stories."

1. Target word: (EN) coal, (BA) ikatz, (SP) carbón - Condition: Concrete | Infrequent

Definition (BA): Sukaldean edo energia sortzeko erabiltzen den substantzia edo material solido eta beltza, materia organikoaren errekuntzaren ondorioz, sarritan Olentzerorekin lotuta.

Definition (SP): Substancia o material sólida y negra utilizada para cocinar o generar energía, resultado de la combustión de materia orgánica, frecuentemente asociada a Olentzero.

"Solid, black substance or material used for cooking or generating energy, resulting from the combustion of organic matter, often associated with Olentzero."

1. Target word: (EN) vapor, (BA) lurrun, (SP) vapor - Condition: Concrete | Infrequent

Definition (BA): Ura asko berotzen denean eta likidoa izan beharrean gasa bihurtzen denean sortzen den elementua.

Definition (SP): Elemento en el que se convierte el agua cuando se calienta mucho y en vez de ser liquida se hace gas.

"Element into which water turns when it is heated a lot and, instead of being liquid, it becomes gas."

1. Target word: (EN) bride, (BA) emaztegai, (SP) novia - Condition: Concrete | Infrequent

Definition (BA): Ezkontza-egunean edo ezkontza-zeremonian soineko zuri berezi bat daraman emakumeari buruz esaten da.

Definition (SP): Se dice de la mujer comprometida que lleva un vestido blanco especial el dia de su boda o ceremonia nupcial.

"A woman who is engaged and wears a special white dress on her wedding day or nuptial ceremony."

1. Target word: (EN) permission, (BA) baimen, (SP) permiso - Condition: Abstract | Frequent

Definition (BA): Zerbait egiteko edo zerbait gertatzeko autorizazioa.

Definition (SP): Autorización para hacer algo o para que algo suceda.

"Authorization to do something or for something to happen."

1. Target word: (EN) explanation, (BA) azalpen, (SP) explicación - Condition: Abstract | Frequent

Definition (BA): Gairen bat argitzea, besteek ulertzeko.

Definition (SP): Aclaración de algún tema para que otros puedan entender.

"Clarification of a topic so that others can understand."

1. Target word: (EN) failure, (BA) porrot, (SP) fracaso - Condition: Abstract | Frequent

Definition (BA): Saiatzen zaren zerbaitetan arrakastarik ez izatea.

Definition (SP): Falta de éxito en algo que se intenta.

"Lack of success in something that is attempted."

1. Target word: (EN) meaning, (BA) esanahi, (SP) significado - Condition: Abstract | Frequent

Definition (BA): Zerbaiten semantika, adibidez, hiztegia erabiltzen dugu hitzena aurkitzeko.

Definition (SP): La semántica de algo, por ejemplo usamos el diccionario para encontrarlo de las palabras.

"The meaning of something, for example, we use the dictionary to find the meaning of words."

1. Target word: (EN) faith, (BA) eskaintza, (SP) oferta - Condition: Abstract | Frequent

Definition (BA): Zerbaiten prezio murriztua iragartzen duen kartela.

Definition (SP): Cartel que anuncia el precio reducido de algo.

"Sign that announces the reduced price of something."

1. Target word: (EN) feeling, (BA) sentimendu, (SP) sentimiento - Condition: Abstract | Frequent

Definition (BA): Bihotzari lotutako egoera emozionala, alaitasuna, tristura, haserrea edo frustrazioa bezalako erreakzioak islatuz.

Definition (SP): Estado emocional vinculado al corazón, reflejando reacciones como alegría, tristeza, enfado o frustración.

"Emotional state related to the heart, reflecting reactions such as joy, sadness, anger, or frustration."

1. Target word: (EN) minimum, (BA) gutxieneko, (SP) mínimo - Condition: Abstract | Frequent

Definition (BA): Ahalik eta kantitate edo maila txikiena, normalean oinarrizko muga gisa ezarria.

Definition (SP): La menor cantidad o grado posible, habitualmente establecido como un límite inferior o básico.

"The smallest amount or degree possible, usually set as a lower or basic limit."

1. Target word: (EN) knowledge, (BA) fede, (SP) fe - Condition: Abstract | Frequent

Definition (BA): Frogarik gabeko zerbaitetan sinestea, hala nola erlijioan sinesten duen jendeak duena.

Definition (SP): Creencia en algo sin necesidad de pruebas, como lo que tiene la gente que cree en un religión.

"Belief in something without the need for proof, like what people who believe in a religion have."

1. Target word: (EN) limit, (BA) muga, (SP) frontera - Condition: Abstract | Frequent

Definition (BA): Bi herrialde edo lurralde banatzen dituen lerroa.

Definition (SP): Línea que divide dos países o territorios.

"Line that divides two countries or territories."

1. Target word: (EN) mistake, (BA) akats, (SP) error - Condition: Abstract | Frequent

Definition (BA): Ekintza, iritzi, erabaki edo emaitzetan zerbait gaizki egitearen ekintza edo hutsegitea.

Definition (SP): Acción o estado de equivocación en la que algo se hace de manera incorrecta o inexacta, ya sea en acciones, juicios, decisiones o resultados.

"Action or state of being wrong in which something is done incorrectly or inaccurately, whether in actions, judgments, decisions, or results."

1. Target word: (EN) choice, (BA) hautaketa, (SP) elección - Condition: Abstract | Frequent

Definition (BA): Politikan kargu publikoak izendatzerako orduan egiten den bezala, bi aukera edo pertsona baino gehiagoren artean bat leheneztearen ekintza.

Definition (SP): Acto de escoger entre dos o más opciones o personas, como se realiza en política para designar cargos públicos.

"Act of choosing between two or more options or people, as is done in politics to appoint public officials."

1. Target word: (EN) context, (BA) testuinguru, (SP) contexto - Condition: Abstract | Frequent

Definition (BA): Gertaera, ideia edo egoera baten inguruko ezaugarri eta baldintzak, hura ulertzeko edo interpretatzeko eragina izan dezaketenak.

Definition (SP): Circunstancias y condiciones que rodean a un evento, idea o situación, y que pueden influir en su comprensión o interpretación.

"Circumstances and conditions surrounding an event, idea, or situation, which can influence its understanding or interpretation."

1. Target word: (EN) religion, (BA) erlijio, (SP) religión - Condition: Abstract | Frequent

Definition (BA): Sineste sistema bat, izaki goren batekin zerikusia duena. Katolikoa, judua edo musulmana dira horren adibide.

Definition (SP): Un sistema de creencias que tiene que ver con un ser supremo. la catolica, la judia o la musulmana son ejemplos.

"A system of beliefs related to a supreme being. Catholicism, Judaism, or Islam are examples."

1. Target word: (EN) hope, (BA) itxaropen, (SP) esperanza - Condition: Abstract | Frequent

Definition (BA): Etorkizunarekiko espero positiboaren sentimendua, bereziki une zailetan. Galtzen den azkena dela esaten da.

Definition (SP): Sentimiento de expectativa positiva hacia el futuro, especialmente en momentos difíciles. Lo último que se pierde.

"Feeling positive expectations towards the future, especially in difficult times. The last thing you lose."

1. Target word: (EN) Monday, (BA) astelehen, (SP) lunes - Condition: Abstract | Frequent

Definition (BA): Asteko lehen eguna, igandearen ostean.

Definition (SP): Primer día de la semana, después del domingo.

"First day of the week, after Sunday."

1. Target word: (EN) respect, (BA) errespetu, (SP) respeto - Condition: Abstract | Frequent

Definition (BA): Bere estatusa, lorpenak edo tasunak direla-eta norbaitekiko edo zerbaitekiko mirespen eta begirune sentimendua.

Definition (SP): Sentimiento de admiración y consideración hacia alguien o algo debido a su estatus, logros o cualidades.

"Feeling of admiration and respect towards someone or something due to their status, achievements, or qualities."

1. Target word: (EN) trust, (BA) konfiantza, (SP) confianza - Condition: Abstract | Frequent

Definition (BA): Norbaiten inguruan eroso eta seguru egotearen sentimendua, zure sekretuak kontatu ahal dizkiozula dakizunean bezala.

Definition (SP): Sentimiento de sentirse cómodo y seguro alrededor de alguien, como cuando sabes que puedes contarle tus secretos.

"Feeling of comfort and security around someone, like when you know you can share your secrets with them."

1. Target word: (EN) risk, (BA) arrisku, (SP) riesgo - Condition: Abstract | Frequent

Definition (BA): Ekintza edo erabaki bat hartzean kalte edo galera bat egotearen aukera, finantza-inbertsioetan, osasun-erabakietan edo muturreko jardueretan egoten dena, adibidez.

Definition (SP): Posibilidad de enfrentar daño o pérdida al realizar una acción o decisión, común en contextos como inversiones financieras, decisiones de salud o actividades extremas.

"Possibility of facing harm or loss when making a decision or taking an action, common in contexts such as financial investments, health decisions, or extreme activities."

1. Target word: (EN) luck, (BA) zorte, (SP) suerte - Condition: Abstract | Frequent

Definition (BA): Arrakasta edo porrota erabakitzen duen eta giza kontrolaz haratagoko indarrei lotutako elementua. Adibidez, azterketa batean arrakasta izan nahi duzunean asko ikasi gabe, edo dado bat bota eta emaitza ona espero duzunean.

Definition (SP): Palabra que refiere a fuerzas más allá del control humano que determinan el éxito o el fracaso, que se usa por ejemplo cuando deseas tener éxito en un examen sin haber estudiado mucho, o cuando tiras un dado y esperas un buen resultado.

"Word referring to forces beyond human control that determine success or failure, used for example when you hope to succeed in an exam without much studying, or when you roll a dice and wish for a good result."

1. Target word: (EN) concept, (BA) kontzeptu, (SP) concepto - Condition: Abstract | Frequent

Definition (BA): Gai baten ideia edo nozio abstraktua, horren kategoria edo ulermen orokorra adierazten duena.

Definition (SP): Idea o noción abstracta de algun tema que representa la categoría de ello, o su comprensión general.

"Abstract idea or notion of a subject that represents its category, or its general understanding."

1. Target word: (EN) association, (BA) elkarte, (SP) asociación - Condition: Abstract | Frequent

Definition (BA): Pertsona edo interes komunak dituen taldea, mota desberdinetakoa izan daitekeena, hala nola kulturala, kiroletakoa edo gurasoena.

Definition (SP): Grupo de personas con intereses comunes, que puede ser de distintos tipos, como cultural, deportiva, o de padres y madres.

"Group of people with common interests, which can be of various types, such as cultural, sports, or parent groups."

1. Target word: (EN) difference, (BA) alde, (SP) diferencia - Condition: Abstract | Frequent

Definition (BA): Gauza bat eta bestea bereizten dituen ezaugarria, edo elementu konparatuen arteko desberdintasuna.

Definition (SP): Característica que distingue una cosa de otra o la disparidad entre elementos comparados.

"Characteristic that distinguishes one thing from another or the disparity between compared elements."

1. Target word: (EN) experience, (BA) esperientzia, (SP) experiencia - Condition: Abstract | Frequent

Definition (BA): Bizitzan zehar gertatutakoen bidez edo ekitaldietan parte hartuz lortutako ezagutza.

Definition (SP): Conocimiento adquirido a través de los acontecimientos de la vida o la participación en eventos.

"Knowledge gained through life events or participation in activities."

1. Target word: (EN) desire, (BA) desira, (SP) deseo - Condition: Abstract | Frequent

Definition (BA): Irrikan dagoen zerbaiterako bultzada, sinbolikoki izar iheskor bati eskatzen zaionako.

Definition (SP): Impulso hacia algo anhelado, comúnmente simbolizado por el acto de pedir a una estrella fugaz, impulsando acciones y la consecución de objetivos.

"Impulse towards something desired, commonly symbolized by the act of wishing upon a shooting star, driving actions and the achievement of goals."

1. Target word: (EN) existence, (BA) existentzia, (SP) existencia - Condition: Abstract | Frequent

Definition (BA): Benetakoa izatearen egoera eta mundu fisikoan, ezerezaren edo absentziaren aurkako egoera.

Definition (SP): Estado de ser real y estar presente en el mundo físico, lo opuesto a la nada o la ausencia.

"State of being real and present in the physical world, the opposite of nothingness or absence."

1. Target word: (EN) soul, (BA) arima, (SP) alma - Condition: Abstract | Frequent

Definition (BA): Izaera espirituala irudikatzen duen entitate ez-materiala, hilezkorra eta gizabanako bakoitzaren bereizgarritzat hartzen dena, filosofia eta erlijio askoren ardatz.

Definition (SP): Entidad inmaterial que representa la esencia espiritual, considerada inmortal y distintiva de cada individuo, central en muchas filosofías y religiones.

"Immaterial entity that represents the spiritual essence, considered immortal and distinctive of each individual, central to many philosophies and religions."

1. Target word: (EN) god, (BA) jainko, (SP) dios - Condition: Abstract | Frequent

Definition (BA): Erlijio monoteistetan izaki gorenena dela esaten da.

Definition (SP): Se dice del ser supremo en religiones monoteístas.

"Referring to the supreme being in monotheistic religions."

1. Target word: (EN) health, (BA) osasun, (SP) salud - Condition: Abstract | Frequent

Definition (BA): Pertsona bat gaixotasun edo gaitzik gabe dagoenean ematen den ongizate fisiko, mental eta sozialaren egoera.

Definition (SP): Estado de bienestar físico, mental y social en el que una persona se encuentra libre de enfermedades y dolencias.

"State of physical, mental, and social well-being in which a person is free from illness and ailments."

1. Target word: (EN) quality, (BA) kalitate, (SP) calidad - Condition: Abstract | Frequent

Definition (BA): Produktu edo zerbitzu baten bikaintasuna definitzen duten ezaugarrien multzoa, sarritan kostu handiagoarekin lotua.

Definition (SP): Conjunto de características que definen la excelencia de un producto o servicio, a menudo asociada con un costo mayor.

"Set of characteristics that define the excellence of a product or service, often associated with a higher cost."

1. Target word: (EN) doubt, (BA) zalantza, (SP) duda - Condition: Abstract | Frequent

Definition (BA): Gai edo gertaera baten inguruko egiaz edo ziurtasunaz konbentzituta ez dagoenaren edo hainbat iritziren artean erabaki ezinik dagoenaren egoera.

Definition (SP): Estado de quien no está convencido de la verdad o certeza de un asunto o hecho, o vacila entre varias opiniones.

"State of being unconvinced of the truth or certainty of a matter or fact, or wavering between several opinions."

1. Target word: (EN) justice, (BA) justizia, (SP) justicia - Condition: Abstract | Frequent

Definition (BA): Denak modu inpartzial eta bidezkoan tratatzeko printzipioa, bereziki lege sisteman.

Definition (SP): Principio de tratar a todos de manera imparcial y equitativa, especialmente en el sistema legal.

"Principle of treating everyone impartially and fairly, especially in the legal system."

1. Target word: (EN) decision, (BA) erabaki, (SP) decisión - Condition: Abstract | Frequent

Definition (BA): Aukera desberdinak pentsatu eta kontuan hartu ondoren egindako aukeraketa edo zehaztapena, askotan ebazpen irmo bat inplikatuz.

Definition (SP): Elección o determinación tomada después de pensar y considerar diferentes opciones, a menudo implicando una resolución firme.

"Choice or determination made after thinking and considering different options, often involving a firm resolution."

1. Target word: (EN) attention, (BA) arreta, (SP) atención - Condition: Abstract | Frequent

Definition (BA): Adimena objektu edo ataza batean kontzentratzea, beste distrakzio batzuk baztertuz. Eskolan despistatzen diren haurrek oso eskasa izaten dute.

Definition (SP): Concentración de la mente en un objeto o tarea, descartando otras distracciones. Los niños que se despistan en clase suelen tener un deficit de ella.

"Concentration of the mind on an object or task, dismissing other distractions. Children who daydream in class often have a deficiency of it."

1. Target word: (EN) purpose, (BA) helburu, (SP) objetivo - Condition: Abstract | Frequent

Definition (BA): Norbaitek lortu nahi duen xede edo estrategia zehatza, sarritan buruan plan edo estrategia bat hartuta.

Definition (SP): Meta o propósito específico que alguien se esfuerza por lograr, a menudo con un plan o estrategia en mente.

"Specific goal or purpose that someone strives to achieve, often with a plan or strategy in mind."

1. Target word: (EN) need, (BA) behar, (SP) necesidad - Condition: Abstract | Frequent

Definition (BA): Biziraupenerako edo ongizaterako funtsezkoa edo oso garrantzitsua den zerbait beharrezoa den egoera. Ingenioa areagotzen duela esaten da.

Definition (SP): Situación en la que se requiere algo esencial o muy importante para la supervivencia o bienestar. Se dice que agudiza el ingénio.

"Situation in which something essential or very important is required for survival or well-being. It is said to sharpen ingenuity."

1. Target word: (EN) interest, (BA) interes, (SP) interés - Condition: Abstract | Frequent

Definition (BA): Zerbaitekiko edo norbaitekiko joera edo begirunea, askotan jasoko den onura edo abantailagatik.

Definition (SP): Tendencia o consideración hacia algo o alguien, muchas veces motivada por el provecho o ventaja que se espera recibir.

"Tendency or consideration towards something or someone, often motivated by the expected benefit or advantage to be gained."

1. Target word: (EN) success, (BA) arrakasta, (SP) éxito - Condition: Abstract | Frequent

Definition (BA): Helburu baten aldeko lorpen edo emaitza positiboa; helburu bat lortu izana.

Definition (SP): Logro o resultado positivo y favorable en relación con una meta; el estado de haber logrado un objetivo.

"Achievement or positive, favorable result in relation to a goal; the state of having accomplished an objective."

1. Target word: (EN) opinion, (BA) iritzi, (SP) opinión - Condition: Abstract | Frequent

Definition (BA): Gai bati buruzko ikuspuntu edo juizio pertsonala, askotan norberaren pertzepzioan eta esperientzian oinarritua; gertaera objektiboekin alderatzen da.

Definition (SP): Punto de vista o juicio personal sobre un tema, a menudo basado en la percepción y la experiencia individual; se contrasta con los hechos objetivos.

"Point of view or personal judgment on a subject, often based on perception and individual experience; contrasted with objective facts."

1. Target word: (EN) nothing, (BA) ezer, (SP) nada - Condition: Abstract | Frequent

Definition (BA): Edozein entitate, objektu edo kopururen absentzia adierazten duen kontzeptua.

Definition (SP): Concepto que denota la ausencia de cualquier entidad, objeto o cantidad.

"Concepto que denota la ausencia de cualquier entidad, objeto o cantidad."

"Concept denoting the absence of any entity, object, or quantity."

1. Target word: (EN) peace, (BA) bake, (SP) paz - Condition: Abstract | Frequent

Definition (BA): Lasaitasuna, harmonia eta gatazkarik eza, bai maila pertsonalean, bai nazioen arteko harremanen testuinguruan.

Definition (SP): Estado de tranquilidad, armonía y ausencia de conflictos, tanto a nivel personal como en el contexto de relaciones entre naciones.

"State of tranquility, harmony, and absence of conflict, both on a personal level and in the context of relations between nations."

1. Target word: (EN) power, (BA) botere, (SP) poder - Condition: Abstract | Frequent

Definition (BA): Beste batzuen erabakietan edo ekintzetan eragiteko gaitasuna; adibidez, nazio bateko errege edo presidente batek duena.

Definition (SP): Capacidad para influir en las decisiones o acciones de otros; por ejemplo, lo que tiene un rey o un presidente de una nación.

"The ability to influence the decisions or actions of others; for example, what a king or president of a nation has."

1. Target word: (EN) truth, (BA) egia, (SP) verdad - Condition: Abstract | Frequent

Definition (BA): Gertaerekiko edo errealitatearekiko adostasuna, gezurrarekin kontrajarrita.

Definition (SP): Conformidad con los hechos o la realidad, en contraposición a la mentira.

"Conformity with facts or reality, as opposed to lies."

1. Target word: (EN) future, (BA) etorkizun, (SP) futuro - Condition: Abstract | Frequent

Definition (BA): Etortzeko dagoen edo oraindik iritsi ez den denbora.

Definition (SP): Tiempo que está por venir o que todavía no ha llegado.

"Time that is yet to come or has not arrived."

1. Target word: (EN) assistance, (BA) laguntza, (SP) ayuda - Condition: Abstract | Frequent

Definition (BA): Beste pertsona bati zerbitzua, baliabideak edo denbora borondatez eskaintzea, egoera zail edo egunerokoetan euskarria emanez.

Definition (SP): Ofrecimiento voluntario de servicio, recursos o tiempo para beneficiar a otra persona, proporcionando soporte en situaciones difíciles o cotidianas.

"Voluntary offering of service, resources, or time to benefit another person, providing support in difficult or everyday situations."

1. Target word: (EN) love, (BA) maitasun, (SP) amor - Condition: Abstract | Frequent

Definition (BA): Emozio sakona, atxikimendu eta lotura pertsonal handia dakarrena, askotan kontuan hartutako ekintzen bidez eta besteekiko arreta handiz adierazia.

Definition (SP): Profunda emoción que implica un gran apego y conexión personal, a menudo expresada a través de acciones consideradas y cuidados atentos hacia otros.

"Deep emotion that involves a strong attachment and personal connection, often expressed through thoughtful actions and attentive care towards others."

1. Target word: (EN) inability, (BA) ezgaitasun, (SP) inhabilidad - Condition: Abstract | Infrequent

Definition (BA): Baldintza negatiboa, "ez-" aurrizkiak adierazten duena, berariazko lan bat eraginkortasunez egiteko behar den ahalmen eza adierazten du.

Definition (SP): Condición negativa, indicada por el prefijo "in-", denota la ausencia de capacidad o aptitud requerida para desempeñar una tarea específica con eficiencia.

"Negative condition, indicated by the prefix 'in-', denoting the absence of the ability or aptitude required to perform a specific task efficiently."

1. Target word: (EN) enchantment, (BA) sorginkeria, (SP) encantamiento - Condition: Abstract | Infrequent

Definition (BA): Malefizio magikoa, Aurora printzesa Loti Ederrean loaldi luze batera eraman zuena.

Definition (SP): Hechizo mágico que indujo a la princesa Aurora en "La Bella Durmiente" a un sueño prolongado.

"Magical spell that caused Princess Aurora in *Sleeping Beauty* to fall into a prolonged sleep."

1. Target word: (EN) incarnation, (BA) berraragiztatze, (SP) reencarnación - Condition: Abstract | Infrequent

Definition (BA): Heriotzaren ondorengo arima berpizten dela sinestea.

Definition (SP): Creencia en el renacimiento del alma en otro cuerpo tras la muerte.

"Creencia en el renacimiento del alma en otro cuerpo tras la muerte."

"Belief in the rebirth of the soul in another body after death."

1. Target word: (EN) righteousness, (BA) zintzotasun, (SP) honradez - Condition: Abstract | Infrequent

Definition (BA): Ekintza eta portaeretan etikoki zuzena eta bidezkoa izatearen ezaugarria, zuzentasuna erakutsiz.

Definition (SP): Cualidad de ser éticamente recto y justo en acciones y comportamientos, mostrando rectitud.

"Quality of being ethically upright and just in actions and behaviors, showing integrity."

1. Target word: (EN) greed, (BA) zekenkeria, (SP) avaricia - Condition: Abstract | Infrequent

Definition (BA): Aberastasunak eta ondasun materialak pilatzeko gehiegizko nahia eta berekoia.

Definition (SP): Deseo excesivo y egoísta de acumular riquezas y bienes materiales.

"Excessive and selfish desire to accumulate wealth and material goods."

1. Target word: (EN) enjoyment, (BA) gozamen, (SP) disfrute - Condition: Abstract | Infrequent

Definition (BA): Esperientzia atseginetatik eratorritako plazer-sentimendua, hala nola janari zoragarri bat dastatzea edo musika ona entzutea.

Definition (SP): Sentimiento de placer derivado de experiencias gratificantes como saborear una comida exquisita o escuchar buena música.

"Feeling of pleasure derived from gratifying experiences such as savoring exquisite food or listening to good music."

1. Target word: (EN) prophecy, (BA) profezia, (SP) profecía - Condition: Abstract | Infrequent

Definition (BA): Etorkizuneko gertaeren iragarpena, iturri jainkotiar batek errebelatua.

Definition (SP): Vaticinio de eventos futuros revelado por una fuente divina.

"Prediction of future events revealed by a divine source."

1. Target word: (EN) multiplication, (BA) biderketa, (SP) multiplicacion - Condition: Abstract | Infrequent

Definition (BA): Zenbaki bat bere buruari hainbat aldiz batzeko eragiketa matematikoa, "x" zeinuak adierazten duena.

Definition (SP): Operación matemática de sumar un número a sí mismo varias veces, indicado por el signo "x".

"Mathematical operation of adding a number to itself multiple times, indicated by the sign 'x'."

1. Target word: (EN) relativity, (BA) erlatibitate, (SP) relatividad - Condition: Abstract | Infrequent

Definition (BA): Einsteinen teoria fisikoa, espazioa eta denbora guztiontzat beti berdinak ez direla azaltzen duena.

Definition (SP): Teoría física de Einstein que explica cómo el espacio y el tiempo no son siempre los mismos para todos.

"Einstein's physical theory that explains how space and time are not always the same for everyone."

1. Target word: (EN) subtraction, (BA) kenketa, (SP) resta - Condition: Abstract | Infrequent

Definition (BA): Kopuruen arteko desberdintasunak aurkitzeko eragiketa matematikoa, "-" ikurrak adierazten duena.

Definition (SP): Operación matemática para encontrar diferencias entre cantidades, indicado por el símbolo "-".

"Mathematical operation to find differences between quantities, indicated by the symbol '-'."

1. Target word: (EN) moderation, (BA) moderazio, (SP) moderación - Condition: Abstract | Infrequent

Definition (BA): Gehiegikeriak saihestu eta oreka mantentzearen praktika, adibidez, alkohola nola kontsumitu behar den DGTren iragarkien arabera.

Definition (SP): Práctica de evitar excesos y mantener un equilibrio, por ejemplo, cómo hay que consumir alcohol según los anuncios de la DGT.

"Practice of avoiding excess and maintaining balance, for example, how alcohol should be consumed according to DGT advertisements."

1. Target word: (EN) frustration, (BA) frustrazio, (SP) frustración - Condition: Abstract | Infrequent

Definition (BA): Bete gabeko itxaropenengatik etsipen-sentimendua, haurrengan kasketak eragin ditzakeena.

Definition (SP): Sentimiento de desánimo por expectativas no cumplidas, que puede causar berrinches en niños.

"Feeling of discouragement due to unmet expectations, which can cause tantrums in children."

1. Target word: (EN) supervision, (BA) ikuskapen, (SP) supervisión - Condition: Abstract | Infrequent

Definition (BA): Besteen aurrerapena edo portaera behatu eta kontrolatzea, behar bezala gauzatzen dela bermatzeko.

Definition (SP): Acción de observar y controlar el progreso o el comportamiento de otros para garantizar su correcta ejecución.

"Action of observing and controlling the progress or behavior of others to ensure proper execution."

1. Target word: (EN) restriction, (BA) murrizketa, (SP) restricción - Condition: Abstract | Infrequent

Definition (BA): Mugak edo kontrolak ezartzen dituen araua.

Definition (SP): Regla que impone limitaciones o controles. Por ejemplo, limitar el suministro de agua durante algunas horas al día en epoca de sequía.

"Rule that imposes limitations or controls. For example, limiting the water supply for certain hours a day during drought."

1. Target word: (EN) disadvantage, (BA) desabantaila, (SP) desventaja - Condition: Abstract | Infrequent

Definition (BA): Besteekin alderatuta egoera txarragoa.

Definition (SP): Situación menos favorable en comparación con otros.

"Less favorable situation compared to others."

1. Target word: (EN) appreciation, (BA) baliozte, (SP) apreciación - Condition: Abstract | Infrequent

Definition (BA): Zerbaiten edo norbaiten kalitatea, balioa edo garrantzia aitortzea, une zail batean lagun baten dedikazioa baloratzen duzunean bezala.

Definition (SP): Reconocimiento y valoración de la calidad, valor o importancia de algo o alguien, como cuando valoras la dedicación de un amigo en un momento difícil.

"Recognition and appreciation of the quality, value, or importance of something or someone, such as when you value a friend's dedication during a difficult time."

1. Target word: (EN) mentality, (BA) mentalitate, (SP) mentalidad - Condition: Abstract | Infrequent

Definition (BA): Adimenarekin zerikusia duen pertsona baten sineste eta pentsamenduen multzoa, irekia, ideia berriak hartzen dituenean.

Definition (SP): Conjunto de creencias y pensamientos de una persona relacionada con la mente, y calificada como abierta cuando muestra receptividad a nuevas ideas.

"Set of beliefs and thoughts of a person related to the mind and described as open when showing receptiveness to new ideas."

1. Target word: (EN) motivation, (BA) motibazio, (SP) motivación - Condition: Abstract | Infrequent

Definition (BA): Helburuak lortzeko edo beharrak asetzeko bultzada, unibertsitatean sartzeko azterketa batean emaitza ona lortu nahi duzunean bezala.

Definition (SP): Impulso para lograr objetivos o satisfacer necesidades, como cuando quieres lograr un buen resultado en un exámen para poder entrar en la universidad.

"Drive to achieve goals or satisfy needs, such as wanting to achieve a good result on an exam to get into university."

1. Target word: (EN) resurrection, (BA) berpizte, (SP) resurrección - Condition: Abstract | Infrequent

Definition (BA): Hil ondoren bizitzara itzultzea, adibidez, Jesusek gurutziltzatzearen ondoren egin zuen bezala.

Definition (SP): Retorno a la vida después de la muerte, ejemplificado por Jesús después de la crucifixión.

"Return to life after death, exemplified by Jesus after the crucifixion."

1. Target word: (EN) obedience, (BA) obedientzia, (SP) obediencia - Condition: Abstract | Infrequent

Definition (BA): Aginduak betetzen dituenaren ezaugarria, jabearen aginduei erantzuten dien txakurra bezala.

Definition (SP): Cumplir instrucciones o mandatos de autoridad, como un perro que responde a las órdenes de su dueño.

"Following instructions or orders from an authority, like a dog responding to its owner's commands."

1. Target word: (EN) creativity, (BA) sormen, (SP) creatividad - Condition: Abstract | Infrequent

Definition (BA): Ideia originalak eta lan berritzaileak sortzeko gaitasuna; askotan, testuinguru artistikoekin lotuta.

Definition (SP): Capacidad de generar ideas originales y trabajos innovadores; a menudo asociado con contextos artísticos.

"Ability to generate original ideas and innovative works, often associated with artistic contexts."

1. Target word: (EN) manipulation, (BA) manipulazio, (SP) manipulación - Condition: Abstract | Infrequent

Definition (BA): Pertsonengan modu sotil baina engainagarrian eragiteko praktika, norbanako toxikoen portaeretan ohikoa dena onura propioak lortzeko.

Definition (SP): Práctica de influir sutilmente pero de manera engañosa sobre personas, común en comportamientos de individuos tóxicos para obtener beneficios propios.

"Practice of subtly but deceptively influencing people, common in the behavior of toxic individuals to gain personal advantage."

1. Target word: (EN) diplomacy, (BA) diplomazia, (SP) diplomacia - Condition: Abstract | Infrequent

Definition (BA): Herrialdeen artean negoziatzeko artea, harreman baketsuak izateko eta akordio onuragarriak lortzeko.

Definition (SP): Arte de negociar entre países para mantener relaciones pacíficas y alcanzar acuerdos beneficiosos.

"The art of negotiating between countries to maintain peaceful relations and reach mutually beneficial agreements."

1. Target word: (EN) limitation, (BA) mugapen, (SP) limitación - Condition: Abstract | Infrequent

Definition (BA): Ekintza- edo mugimendu-ahalmena murriztea, errepideetan gehienezko abiadura ezartzen denean bezala.

Definition (SP): Disminución de la capacidad de acción o movimiento, como cuando se establecen topes de velocidad en carreteras.

"Reduction in the capacity for action or movement, such as when speed limits are set on roads."

1. Target word: (EN) innovation, (BA) berrikuntza, (SP) innovación - Condition: Abstract | Infrequent

Definition (BA): Produktuetan edo prozesuetan ideia hobetuak aplikatzea.

Definition (SP): Aplicar ideas mejoradas en productos o procesos.

"Applying improved ideas to products or processes."

1. Target word: (EN) superiority, (BA) nagusitasun, (SP) superioridad - Condition: Abstract | Infrequent

Definition (BA): Beste batzuk baino maila altuagoan edo hobean egotea, eta harrokeria- edo goritasun-jarreran ager daitekeena.

Definition (SP): Estar en una posición más alta o mejor que otros, y que puede manifestarse en una actitud de arrogancia o altivez.

"Being in a higher or better position than others, which can manifest as an attitude of arrogance or haughtiness."

1. Target word: (EN) loyalty, (BA) leialtasun, (SP) lealtad - Condition: Abstract | Infrequent

Definition (BA): Etengabe erakutsitako irmotasuna eta fideltasuna, jabea baldintzarik gabe babesten duen txakurrarena bezala.

Definition (SP): Constancia y fidelidad demostrada continuamente, como la de un perro que acompaña y protege a su dueño incondicionalmente.

"Consistency and loyalty demonstrated continuously, like that of a dog that accompanies and protects its owner unconditionally."

1. Target word: (EN) compassion, (BA) gupida, (SP) compasión - Condition: Abstract | Infrequent

Definition (BA): Besteen sufrimenduarekiko enpatia eta ulermen sentimendua, oinazea edo zailtasunak arintzeko neurriak hartzera bultzatzen duena, hala nola, kezka azaltzeratu eta laguntza eskaintzera.

Definition (SP): Sentimiento de empatía y comprensión hacia el sufrimiento de los demás, que impulsa a tomar medidas para aliviar su dolor o dificultades, como mostrar preocupación y ofrecer ayuda.

"A feeling of empathy and understanding toward the suffering of others, which drives actions to alleviate their pain or difficulties, such as showing concern and offering help."

1. Target word: (EN) legacy, (BA) legatu, (SP) legado - Condition: Abstract | Infrequent

Definition (BA): Kontribuzioek eremu jakin batean duten eragin iraunkorra; esate baterako, idazleen eta irakurleen ondorengo belaunaldiei inspirazioa ematen dien autoreren baten literatura-lanak.

Definition (SP): Impacto duradero de contribuciones en un campo específico, como las obras literarias de alguna autora que inspiran a generaciones posteriores de escritores y lectores.

"The lasting impact of contributions in a specific field, such as the literary works of an author that inspire subsequent generations of writers and readers."

1. Target word: (EN) raw, (BA) gordin, (SP) crudo - Condition: Abstract | Infrequent

Definition (BA): Berezko egoeran edo prozesatu gabe dagoen zerbait, janaria prestatu edo findu aurretik egoten den bezala.

Definition (SP): Algo en su estado natural o no procesado, como en referencia a alimentos antes de haber sido cocinado o refinados.

"Something in its natural or unprocessed state, as in reference to food before it has been cooked or refined."

1. Target word: (EN) relevance, (BA) garrantzi, (SP) relevancia - Condition: Abstract | Infrequent

Definition (BA): Testuinguru jakin batean zerbaitek duen nabarmentasun- edo egokitasun-maila.

Definition (SP): Grado de importancia o pertinencia que algo tiene en un contexto específico.

"The degree of importance or relevance that something has in a specific context."

1. Target word: (EN) attraction, (BA) atrakzio, (SP) atracción - Condition: Abstract | Infrequent

Definition (BA): Interesa, atsegina edo desioa sortzen duen eragina, hala nola pertsonen arteko sexu-bulkada edo imanek elkarren artean duten indarra.

Definition (SP): Influencia que genera interés, agrado o deseo, como el impulso sexual entre personas o la fuerza que ejercen los imanes entre sí.

"Influence that generates interest, pleasure, or desire, such as sexual attraction between people or the force magnets exert on each other."

1. Target word: (EN) socialism, (BA) sozialismo, (SP) socialismo - Condition: Abstract | Infrequent

Definition (BA): Berdintasun ekonomikoa lortzeko, estatuak ekonomia zuzentzen duen sistema, kapitalismoarekin kontrajarrita, baina komunismoan baino lehunago.

Definition (SP): Sistema donde el estado dirige la economía para igualdad económica, en contraposición al capitalismo pero menos extremo que el comunismo.

"A system where the state directs the economy to achieve economic equality, contrasting with capitalism but less extreme than communism."

1. Target word: (EN) goodness, (BA) jainkosa, (SP) bondad - Condition: Abstract | Infrequent

Definition (BA): Eskuzabala eta ona izatea besteekiko tratuan.

Definition (SP): Ser generoso y bueno en el trato con los demás.

"Being generous and kind in dealing with others."

1. Target word: (EN) excellence, (BA) bikaintasun, (SP) excelencia - Condition: Abstract | Infrequent

Definition (BA): Kalitate edo lan maila handia, ohikoa ikerketa nabarmenean edo emaitza akademiko hobeenetan.

Definition (SP): Alto nivel de calidad o desempeño, habitual en la investigación destacada o los resultados académicos más sobresalientes.

"A high level of quality or performance, typical of outstanding research or the most exceptional academic results."

1. Target word: (EN) sensitivity, (BA) sentikortasun, (SP) sensibilidad - Condition: Abstract | Infrequent

Definition (BA): Estimulu garrantzitsuen aurrean erantzun emozional sendoak, hala nola malkoak artearen, musikaren edo giza izaeraren lekukotzaren aurrean.

Definition (SP): Respuestas emocionales intensas ante estímulos significativos tales como lágrimas ante el arte, la música o el testimonio de la condición humana.

"Intense emotional responses to significant stimuli, such as tears evoked by art, music, or testimony to the human condition."

1. Target word: (EN) inspiration, (BA) inspirazio, (SP) inspiración - Condition: Abstract | Infrequent

Definition (BA): Pertsona bat ideia esanguratsuak sortzera bultzatzen duen sormen-estimulua, hala nola artista bat maisulan bat sortzera motibatzen duen hasierako txinparta.

Definition (SP): Estímulo creativo que impulsa a una persona a generar ideas significativas, como la chispa inicial que motiva a un artista a crear una obra maestra

"A creative stimulus that drives a person to generate meaningful ideas, like the initial spark that motivates an artist to create a masterpiece."

1. Target word: (EN) renewal, (BA) berritze, (SP) renovación - Condition: Abstract | Infrequent

Definition (BA): Zerbait zaharra eguneratzea edo hobetzea, adibidez, etxe zahar bat birmoldatzea.

Definition (SP): Actualizar o mejorar algo antiguo, como la remodelación de una casa antigua.

"To update or improve something old, like the renovation of an old house."

1. Target word: (EN) continuity, (BA) jarraitasun, (SP) continuidad - Condition: Abstract | Infrequent

Definition (BA): Ekintza edo egoera bat etengabe mantentzea, hala nola tratamendu medikoetan konstantzia izatea.

Definition (SP): Mantener una acción o situación de forma constante, como la constancia en tratamientos médicos.

"To maintain an action or situation consistently, such as adherence to medical treatments."

1. Target word: (EN) elimination, (BA) ezabaketa (kanporatzea?), (SP) eliminación - Condition: Abstract | Infrequent

Definition (BA): Zerbait edo norbait baztertzea edo mugitzea, jokalari bat joko batetik ateratzearen edo artxibo digital bat kentzearen baliokidea.

Definition (SP): Desechar o remover algo o a alguien, equivalente a sacar un jugador de un juego o suprimir un archivo digital.

"To discard or remove something or someone, equivalent to taking a player out of a game or deleting a digital file."

1. Target word: (EN) madness, (BA) eromen, (SP) locura - Condition: Abstract | Infrequent

Definition (BA): Egoera psikikoa, arrazoirik gabe, ohiz kanpoko jarrera eta iritziekin, Kixotek bizi zuen antzekoa haize-erroten kontra borrokan.

Definition (SP): Condición psíquica con pérdida de la razón con conductas y juicios inusuales, similar a Don Quijote luchando contra molinos de viento.

"Mental condition involving a loss of reason, with unusual behaviors and judgments, akin to Don Quixote fighting windmills."

1. Target word: (EN) pride, (BA) harrotasun, (SP) orgullo - Condition: Abstract | Infrequent

Definition (BA): Norberaren lorpenengatik edo talde bateko kide izateagatik gogobetetzea, LGBTI komunitateko ekitaldietan ospatzen den bezala.

Definition (SP): Satisfacción por logros personales o pertenencia a un grupo, como el que se celebra en eventos de la comunidad LGTBI.

"Satisfaction from personal achievements or belonging to a group, such as that celebrated in LGBTQ+ community events."

1. Target word: (EN) tact, (BA) ukimen, (SP) tacto - Condition: Abstract | Infrequent

Definition (BA): Kontaktu fisikoaren bidez objektuak edo gauzak hauteman eta bereizteko bost zentzumenetako bat.

Definition (SP): Uno de los cinco sentidos que permite percibir y distinguir los objetos o las cosas a través del contacto físico.

"One of the five senses that allows the perception and distinction of objects or things through physical contact."

1. Target word: (EN) advancement, (BA) aurrerapen, (SP) avance - Condition: Abstract | Infrequent

Definition (BA): Ezagutzaren jarduera edo eremu batean aurrera egitea, geldialdiaren edo atzerapenaren kontrakotzat hartuta.

Definition (SP): Progreso en una actividad o campo del conocimiento, considerado como lo contrario al estancamiento o retroceso.

"Progress in an activity or field of knowledge, considered the opposite of stagnation or regression."

1. Target word: (EN) liberation, (BA) askapen, (SP) liberación - Condition: Abstract | Infrequent

Definition (BA): Gatibu edo giltzapean dagoen norbaiten egoerari amaiera ematen dion prozesua, bahituta edo preso baten kasuan bezala.

Definition (SP): Proceso que da fin al estado de alguien en cautividad o reclusión, como en el caso de un secuestrado o un preso.

"Process that brings an end to someone's state of captivity or imprisonment, as in the case of a kidnapped person or a prisoner."
