# Supplementary material for: Basque-Spanish Bilingual Aphasia: A Case-Study to Reveal Frequency-Based, Language-Agnostic Lexical Organization in Bilinguals
Source: Neurobiol Lang (Camb). 2025 Jun 23;6:nol_a_00170. doi: 10.1162/nol_a_00170 (PMC12208705; doi:10.1162/nol_a_00170)
Supplement: Supplementary file 4 [file nol-6-1-170-s004.docx]

# Supplementary Materials 3. Detailed Results of the Bilingual Aphasia Test (Basque-Spanish Adaptation)

Summary of the participant’s performance on the Bilingual Aphasia Test (Basque-Spanish Adaptation) (Paradis, 1987; Erriondo, Álvarez, & Bidegain, 1989). The table presents raw scores out of the total number of items, followed by the percentage of correct responses for each language (Basque/Spanish). The assessment includes tasks evaluating comprehension, production, reading, writing, repetition, and translation abilities in both languages. Percentages indicate accuracy in each task relative to the total number of items.

| Test | Basque  (Correct/Total, %) | Spanish  (Correct/Total, %) |
| --- | --- | --- |
| **Bilingual Aphasia Test (BAT) – Part B** |  |  |
| Comprehension |  |  |
| Pointing | 10/10 (100%) | 10/10 (100%) |
| Simple and Semi-complex Commands | 9/10 (90%) | 9/10 (90%) |
| Complex Commands | 4/5 (80%) | 5/5 (100%) |
| Auditory Verbal Discrimination | 15/18 (83%) | 17/18 (94%) |
| Syntactic Comprehension | 86/87 (99%) | 85/87 (98%) |
| Auditory Comprehension (Paragraph) | 3/5 (60%) | 5/5 (100%) |
| Grammaticality Judgments | 10/10 (100%) | 10/10 (100%) |
| Semantic Acceptability | 10/10 (100%) | 10/10 (100%) |
| Lexical Decision |  |  |
| Lexical Acceptability (Words) | 19/20 (95%) | 20/20 (100%) |
| Lexical Acceptability (Non-words) | 9/10 (90%) | 10/10 (100%) |
| Semantic Categories | 5/5 (100%) | 5/5 (100%) |
| Production |  |  |
| Derivational Morphology | 8/10 (80%) | 10/10 (100%) |
| Morphological Opposites | 10/10 (100%) | 10/10 (100%) |
| Naming | 7/20 (35%) | 9/20 (45%) |
| Semantic Opposites | 6/10 (60%) | 8/10 (80%) |
| Synonyms | 5/5 (100%) | 5/5 (100%) |
| Antonyms | 9/10 (90%) | 10/10 (100%) |
| Sentence Construction | 14/15 (93%) | 15/15 (100%) |
| Automatic Series | 3/3 (100%) | 3/3 (100%) |
| Mental Calculation | 15/15 (100%) | 14/15 (93%) |
| Reading |  |  |
| Words (Aloud) | 10/10 (100%) | 10/10 (100%) |
| Sentences (Aloud) | 10/10 (100%) | 10/10 (100%) |
| Word-Picture Matching | 10/10 (100%) | 10/10 (100%) |
| Sentence-Picture Matching | 10/10 (100%) | 10/10 (100%) |
| Paragraph (Silent) + Comprehension | 6/6 (100%) | 4/6 (67%) |
| Writing |  |  |
| Copying | 5/5 (100%) | 5/5 (100%) |
| Dictation (Words) | 5/5 (100%) | 5/5 (100%) |
| Dictation (Sentence) | 4/5 (80%) | 5/5 (100%) |
| Repetition |  |  |
| Words | 20/20 (100%) | 20/20 (100%) |
| Non-words | 10/10 (100%) | 10/10 (100%) |
| Sentences | 7/7 (100%) | 7/7 (100%) |
| **Bilingual Aphasia Test (BAT) – Part C** |  |  |
| Written Word Matching (Basque → Spanish) | 5/5 (100%) |  |
| Written Word Matching (Spanish → Basque) | 5/5 (100%) |  |
| Translation of Words (Basque → Spanish) | 3/10 (30%) |  |
| Translation of Words (Spanish → Basque) | 8/10 (80%) |  |
| Translation of Sentences (Basque → Spanish) | 17/18 (94%) |  |
| Translation of Sentences (Spanish → Basque) | 14/18 (78%) |  |
| Grammaticality Judgment (Basque) | 7/8 (88%) |  |
| Grammaticality Judgment (Spanish) | 8/8 (100%) |  |
| Correction of Agrammatical Sentences (Basque) | 7/8 (88%) |  |
| Correction of Agrammatical Sentences (Spanish) | 8/8 (100%) |  |
